# Zeptomole Imaging of Cytosolic MicroRNA Cancer Biomarkers with A Light-Controlled Nanoantenna

**DOI:** 10.1007/s40820-021-00732-1

**Published:** 2021-10-21

**Authors:** Yang Song, Xiaoli Cai, Grayson Ostermeyer, Shichao Ding, Dan Du, Yuehe Lin

**Affiliations:** 1 School of Mechanical and Materials Engineering, Washington State University, Pullman, WA 99164 USA; 2 Nanosong Systems LLC, Redmond, WA 98052 USA; 3 School of Biological Sciences, Washington State University, Pullman, WA 99164 USA

**Keywords:** Light-harvest, Nanoantenna, miRNA detection, Zeptomole, Multiple cell lines

## Abstract

Detecting and quantifying intracellular microRNAs (miRNAs) are a critical step in resolving a cancer diagnostic and resolving the ensemble of gene products that orchestrate the living state of cells. However, the nanoprobe for detecting low abundance miRNAs in cell cytosol is restricted by either the “one-to-one” signal-trigger model or difficulty for cytosol delivery. To address these challenges, we designed a light-harvesting nanoantenna-based nanoprobe, which directs excitation energy to a single molecule to sensitively detect cytosolic miRNA. With light irradiation, the light-harvesting nanoantenna effectively disrupted lysosomal structures by generation of reactive oxygen species, substantially achieved cytosol delivery. The nanoantenna containing > 4000 donor dyes can efficiently transfer excitation energy to one or two acceptors with 99% efficiency, leading to unprecedented signal amplification and biosensing sensitivity. The designed nanoantenna can quantify cytosolic miR-210 at zeptomolar level. The fluorescence lifetime of the donor exhibited good relationship with miR-210 concentration in the range of 0.032 to 2.97 amol/ngRNA. The zeptomole sensitivity of nanoantenna provides accurate bioimaging of miR-210 both in multiple cell lines and in vivo assay, which creates a pathway for the creation of miRNA toolbox for quantitative epigenetics and personalized medicine.

**Supplementary Information:**

The online version contains supplementary material available at 10.1007/s40820-021-00732-1.

## Highlights

- Based on førster resonance energy transfer, the nanoantenna containing over 4000 donor dyes can achieve unprecedented signal amplification and biosensing sensitivity.
- With light irradiation, the light-harvesting nanoantenna effectively disrupted lysosomal structures by generation of reactive oxygen species, substantially achieved cytosol delivery.
- The zeptomolar sensitivity of nanoantenna provides accurate bioimaging of miR-210 in multiple cell lines and in vivo assay, which creates a pathway for the creation of miRNA toolbox for quantitative epigenetics and personalized medicine.

**Supplementary Information:**

The online version contains supplementary material available at 10.1007/s40820-021-00732-1.

## Graphical abstract

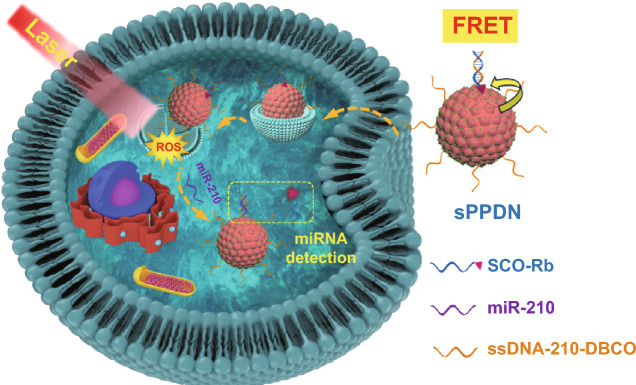

## Introduction

MicroRNAs (miRNAs) are a class of short endogenous noncoding RNAs that selectively hybridize to messenger RNAs (mRNAs), causing direct repression of polypeptide synthesis [1–3]. As key mediators of RNA silencing and post-transcriptional regulation for a wide variety of biological processes, including DNA repair, cell division and apoptosis, intracellular signaling, and cellular metabolism, aberrant expression of miRNAs is implicated in numerous human diseases, such as Alzheimer’s disease, multiple sclerosis, type II diabetes, and breast cancer [4, 5]. Developing new tools to precisely quantify cellular levels of miRNAs, therefore, offers considerable clinical value for the diagnosis and treatment of these diseases. Real-time quantitative PCR (qPCR), the most common technique for assaying cellular miRNA abundance, is an amplification-based strategy that measures miRNA levels using only nanograms of sample RNA [6–9]. This method is convenient for quantifying cellular miRNAs at relatively high abundance, but a more sensitive approach is desirable to resolve miRNA at low expression levels, such as those with a thousand or fewer copies per cell, or in the attomolar range [7, 8]. Alternative techniques for quantifying low expression miRNAs, such as fluorescent probes and electrical-based methods, similarly offer insufficient sensitivity and signal discrimination [10–13] .

Nanoprobe systems involving Førster resonance energy transfer (FRET) were designed and expected to overcome these limitations for miRNAs detection [11–13]. Since “light-harvesting nanoantenna” for dye-loaded NPs was first reported by Klymchenko’s group to amplify the fluorescence of a single dye, fluorescent nanoparticles (FNPs) have been demonstrated to be capable FRET energy donors, which transfer laser-derived excitation energy to a single dye molecule, resulting in average signal amplification [14]. They further used this nanoantenna to develop a FRET-based platform for amplified detection of nucleic acids [15, 16]. In their work, the light-harvesting nanoantenna exhibited superior sensitivity for FRET-based nucleic acids detection to other molecular probes, which also showed potential in FRET assays of miRNAs at very low concentrations. However, FNPs currently available are generally unsuitable to be energy donors for miRNA detection because their particle sizes exceed the normal FRET operating range of 1 to 10 nm to perform effectively [15, 16]. When the abundance of intracellular miRNA is extremely low, these nanoprobes will face the challenge on sensitivity owing to the “one-to-one” signal biosensing model (one miRNA triggers one FNP signal), leading to inadequate sensitivity [17–19]. Hence, controlling the emission of FRET donor by a single miRNA remains a big challenge in the design of ultrasensitive nanoprobes [20–22]. Recently, a light-harvesting nanoantenna-based enzyme-free method was developed for miRNA detection in cellular extracts [23]. Taking advantage of the antenna amplification effect, the nanoprobe demonstrated excellent analytical performance for the detection of miRNA with a limit of detection (LOD) down to 1.3 pM (21 amol).

Furthermore, FRET-based nanoprobes must be delivered to the cytosol before the FNPs can interact with miRNAs, which are primarily in the cytosol to regulate gene expression [24, 25]. Currently, it is an enormous challenge to deliver most FRET-based nanoprobes into targeted cytosol [26, 27]. The release of the nanoprobes from vesicular compartments is highly desired, which not only can avoid enzymatic degradation but also ensure the effective interaction with the targets to the cytosol [28, 29]. Photochemical internalization (PCI), using a combination of photosensitizers and light, has been successfully applied as a novel technology for the release of endocytosed macromolecules into the cytosol by inducing reactive oxygen species (ROS)-mediated damage of the membranes [30]. The basis for PCI is described in detail by Berg’s group, and it shows great potential for site-specific drug delivery and endosomal escape of nanoparticles [31].

To overcome the difficulties of efficient FRET-based miRNA nanoprobes, the designed nanoprobes need to meet the following criteria: (1) the nanoprobes should have bright fluorescence and can transfer whole excitation energy to one or two acceptors [15], (2) it can escape from lysosomes easily, and (3) it can accumulate in the cytosol [32]. Artificial light-harvesting antenna systems can mimic primary step in natural photocatalysis for better utilization of light energy [33, 34]. By loading a small number of acceptors, an artificial light-harvesting antenna system induces the emission enhancement of donor and efficient energy transfer to acceptor, based on the mechanism of FRET [35].

Herein, we describe a novel artificial light-harvesting nanoantenna assembly that is capable of quantifying cytosolic miRNA at zeptomolar concentrations. A polyethylene glycol (PEG) chain coupled to a polyhedral oligomeric silsesquioxane (POSS) is first reacted with a dansyl (DNS) molecule, a hydrophobic fluorophore, to form the PEG-POSS-DNS system. The amphiphilic PEG-POSS-DNS conjugates self-assemble into PEG-POSS-DNS nanoantennae (PPD) through hydrophobic collapse. DNS, the FRET donor, is effectively packed away from each other by POSS molecules in this design, which inhibits the self-quenching of these energy donors. Next, the azide group undergoes a click cycloaddition reaction with dibenzocyclooctyne (DBCO), which is conjugated to a single-strand DNA (ssDNA) encoding a miRNA of interest (denoted as PPDN). The short capture oligonucleotide (SCO) is conjugated with rose bengal (Rb, photosensitizer) and hybridized with the PPDN to form SOC: PPDN (sPPDN). The excitation energy freely migrates from 2000 energy donors to a single acceptor with 99% efficiency, leading to high signal amplification and biosensing sensitivity. The sPPDN can achieve enhanced lysosomal escape based on PCI by increasing ROS generation under laser irradiation. The fluorescence lifetime change of sPPDN toward miRNAs is further analyzed by the fluorescence lifetime imaging microscopy (FLIM), which avoids autofluorescence interference, as well as the susceptibility to light sources, probe concentrations, and environmental differences [36, 37]. The exceptionally low LOD, high sensitivity, and high specificity of this nanoantenna-based biosensing system enable quantitative intracellular miRNA imaging in multiple live cells and live animals with zeptomolar sensitivity.

## Experimental

### Synthesis of PPD

For synthesis of the PPD, 1 mL of the PEG-POSS-DNS/mPEG-POSS/fPEG-POSS/aPEG-POSS polymer mixture (optimized molar ratio: 80:18:1:1) in acetonitrile (2 mg mL^−1^) was added quickly using a micropipette to 9 mL of 20 mM phosphate buffer, pH 7.4 at 4 °C under shaking. The achieved solution was then quickly diluted fivefold with the phosphate buffer. The obtained NPs solution was kept at 4 °C for further use.

### Synthesis of sPPDN

Aliquots of DNA (3 μM ssDNA-210-DBCO) were added to 300 μL of PPD (10 μM). The reaction was mixed and kept overnight at 40 °C in Thermomixer without shaking protected from light. Then, the reaction was cooled down to room temperature. For annealing with SCO-Rb, the aliquot of SCO-Rb with different concentrations was added, the above mixture was heated to 65 °C in a water bath for 3 min. To complete hybridization, the reaction was cooled down to room temperature and kept in the dark for 2 h. Then, the mixture was washed with 20 mM phosphate buffer and purified by centrifugation using centrifuge filters (100 kDa). The obtained sPPDN solution was kept at 4 °C for further use.

## Results and Discussion

### Design and Synthesis of the Nanoantenna Assembly

**Scheme 1:**
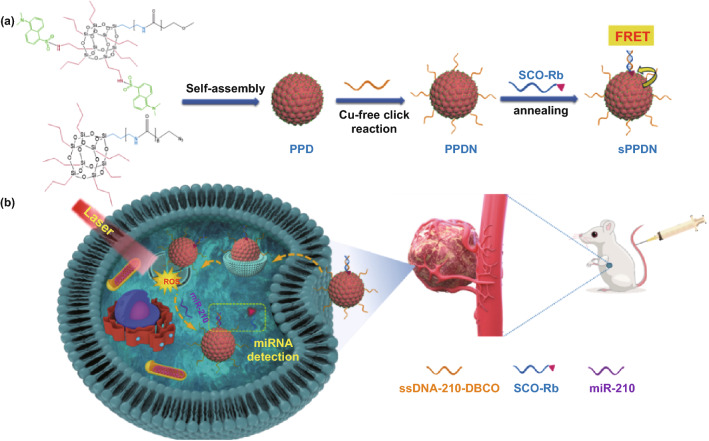
**a** Schematic representation of a section of the self-assembled sPPDN, which contains a large number of donors and only a few acceptors. Initially, the excitation energy is delocalized over many donor molecules (DNS) and then transferred to acceptors (Rb) mainly through exciton migration pathways. **b** The light-triggerable nuclear lysosomal escape strategy using sPPDN in vivo

Fabrication of the nanoantenna begins with the chemical addition of PEG derivatives to a cubic nanocage known as a POSS, a maneuver which positions amine groups at each of POSS’s eight corners for further modification [38]. Hydrophobic DNS dyes are appended to PEG-POSS conjugates at the peripheral amines, resulting in a spatially separated fluorophore arrangement that is ideal for light-harvesting energy transfer reactions [39]. The detailed preparation, purification, and characterizations of amphiphilic PEG-POSS-DNS conjugates are described in the supporting information (Figs. S1 and S2). POSS functions as a structural spacer in this molecular configuration, which prevents DNS interaction, minimizes aggregation-caused quenching (ACQ) and ensures ultrafast excitation energy migration required for efficient light harvesting [40]. The amphiphilic PEG-POSS-DNS conjugates self-assemble into PEG-POSS-DNS nanoantennae in an aqueous solution by hydrophobic collapse. The azide group of the nanoantenna system reacts with the dibenzocyclooctane (DBCO) labels affixed to single-strand oligonucleotide (ssDNA) probes via a click cycloaddition reaction. The segment of nucleotide sequences in the oligo probe reflects the sense strand of DNA coding for the mRNA complement of the miRNA target. The final component of the nanoantenna system is a target competitive nucleotide sequence, the short capture oligonucleotide (SCO), to which Rb is conjugated. Hybridization of SCO-Rb to the nanoantenna generates the PPDN-DBCO-ssDNA: SCO-Rb complex, which we refer to as the sPPDN (Scheme 1). We selected ssDNA-210 to be the sPPDN oligonucleotide probe in our proof-of-concept trials demonstrating the nanoantenna’s efficacy to quantify cytosolic concentrations of the hypoxemic-response miRNA, miR-210.

**Fig. 1:**
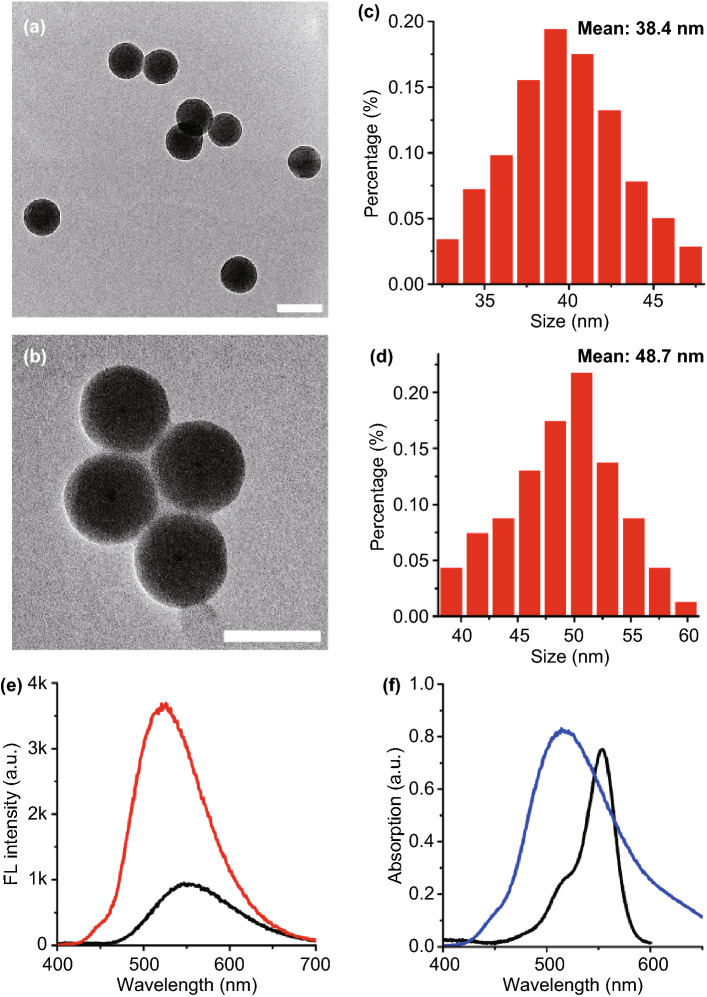
TEM image of **a** PPD and **b** sPPDN. Scale bar: 50 nm. Size distribution of **c** PPD and **d** sPPDN. **e** Fluorescent spectra of PPD (red) in PBS solution and DNS dye (black) in DMSO under 365 nm wavelength light excitation. **f** Fluorescent spectra of PPDN (blue) under 365 nm wavelength excitation and absorption spectra of Rb (black)

We confirmed PPD synthesis and sPPDN formation by transmission electron microscopy (TEM). The PPD assembly remained broadly spherical in shape following azide-alkyne cycloaddition (Fig. 1a). Dynamic light scattering (DLS) measurements showed that PPD has an average hydrodynamic diameter of 38.4 nm in an aqueous solution (Fig. 1c), which increases to 48.7 nm after hybridization to SOC-Rb and enlargement caused by additional hydration layers (Fig. 1b, d). Moreover, the anisotropy value of Rb increased from 0.052 ± 0.002 (free SOC-Rb) to 0.083 ± 0.005, confirming the SOC-Rb/ssDNA-210 hybridization. Zeta potential (ζ-potential) of PPD shifted from 13.1 to -23.7 mV after treatment with SCO-Rb (Fig. S3). This observation confirmed competitive nucleotide sequence interaction with the nanoantenna because the electronegativity of the oligonucleotide sugar-phosphate backbone rendered the electric potential negative.

We then mixed various chemical agents with PPD in an aqueous solution to study the self-assembly mechanism in different chemical environments (Fig. S4). The surfactant Tween-80 effectively reduced the average size of PPD assemblies by disrupting the hydrophobic interactions holding the PEG-POSS-DNS components together. On the other hand, neither the competitive hydrogen bonds present in urea nor the surface charge neutralization potential caused by NaCl, otherwise known as the electrostatic shielding effect, effectively disrupt PPD.

The gel retardation assay was further performed to study the interact affinity of ssDNA-210-DBCO with PPD. A constant amount of ssDNA-210-DBCO (3 μM) was incubated with different concentrations (0–10 μM) of PPD (Fig. S5). A complete electrophoretic shift is observed for naked mRNA. PPD effectively formed complexes with ssDNA-210-DBCO following an increase in PPD concentrations. A complete binding was achieved at PPD concentration of 10 μM, confirming successful adsorption of ssDNA-210-DBCO. SCO-Rb were expected to interact at the PPDN surface. As shown in Fig. 1b, after conjugated with SCO-Rb, larger nano-complexes were formed and detected by TEM. Adsorption of SCO-Rb by PPDN protected SCO-Rb against nuclease degradation, a prerequisite for intracellular biosensing. As shown in Fig. S6, treatment of ssDNA-210-DBCO with DNAse (10 mU) at 20 min and 1 h resulted in complete degradation of ssDNA-210-DBCO. Significant amounts of ssDNA-210-DBCO in the sPPDN complex were still detected after treatment of DNAse even at a longer incubation (1 h), indicating strong protection of ssDNA-210-DBCO on sPPDN against the enzymatic cleavage. Besides, the zeta potential data showed a negligible difference between sPPDN with different interferences (FBS and human serum), indicating good stability (Fig. S7). The stability was further proved by the TEM images of sPPDN in borate buffer (pH = 9.0) and cell culture medium (Fig. S8). As can be seen from Fig. S8, the probes keep stable compared to the one in PBS buffer of Fig. 1b. Moreover, the probes keep the similar size of nanoparticles in different conditions, which indicates that the particles should be stable in buffers of different pH and in cell culture medium (Fig. S9).

### Signal Amplification by PPD

Previous investigations demonstrated that polycyclic aromatic hydrocarbon fluorophores such as DNS exhibit very weak fluorescence when ordered in tightly packed or π-conjugated organic molecules due to ACQ [41]. In POSS systems, fluorophore-linked substituents may be attached to functional Si centers separated by O bridges, providing both sufficient spaces to prevent ACQ and maximal FRET energy donor density to carry out light harvesting effectively. Considering that the DNS/POSS molar ratio is ~ 1.8 and DNS-conjugated PEG derivatives can react with up to eight Si centers, PPD is designed to operate at high quantum efficiency balancing between the competing FNP performance priorities of high energy transfer and minimal ACQ induction. Aqueous solutions containing PPD assemblies emitted stronger fluorescence than those with DNS dyes alone (Fig. 1e), a result attributable to encapsulation of DNS dyes within the nanoantenna and avoidance of DNS π-π stacking. DNS encapsulation also caused a hypsochromic shift in the emission spectrum from 550 to 513 nm, providing further verification of fewer intermolecular dipole–dipole interactions and π–π stacking between DNS molecules in PPD assemblies. In varying DNS conjugate loading ratios from 0 to 100 mol% within PPD, we observed maximal fluorescence intensity at 80 mol% (Fig. S10). Such emission intensity change could be explained by the change of DNS-DNS intermolecular distance within these POSS structures. When the density of orderly aligned DNS molecules is low, the DNS-DNS distance is large enough to prevent any potential fluorescence quenching induced by π–π interactions, thus the emission intensity increased proportionally to the DNS density. Interestingly, the self-assembly of PPD significantly increased the fluorescence quantum yield (QY) (Fig. S11). Compared to the QY of DNS (10.4%), the QY of PPD showed a significant increase in varying DNS conjugate loading ratios, indicating that the tuning of DNS density within the PPD enabled a fine tuning of the QY. As shown in Fig. S11, PPD (80 mol%) exhibited the highest QY of 47%. The importance of self-assembly of PPD in the fluorescence properties was further revealed by studying the excited-state dynamics of these materials. For that, we performed fluorescence lifetime (τ) measurements (Table S2). Before the self-assembly, DNS in aqueous solution exhibited a double exponential decay, with a short-lived decay component (τ_1_) of 12.5 ns (relative contribution of the lifetime component for τ_1_: α_1_ **~** 59%) and a long-lived decay component (τ_2_) of 18.7 ns (relative contribution of the lifetime component for τ_2_: α_2_ **~** 41%). This result is in good agreement with the corresponding low QY due to the appearance of the high percentage of short-lived decay components. In contrast to DNS, self-assembled crystalline PPD showed a significant decrease in τ_1_ and α_1_ due to the reduced self-quenching of DNS molecules. Among them, PPD (80 mol%) exhibited the low τ_1_ and showed a high α_2_ of over 96% compared to that of DNS (~ 41%), suggesting that the contribution of the long-lived decay time to the emission remained dominant and the emission enhancement originated from the decrease of  π–π interactions (Table S2). We further determined the radiative (*k*_r_) and nonradiative (*k*_nr_) decay rate constants based on the ratio of the values of QY to τ. As shown in Table S3, PPD (80 mol%) exhibited the smaller *k*_nr_ value compared to that of DNS, confirming the significant impact of PPD on the suppression of nonradiative decay. In an aqueous solution, PPD (80 mol%) exhibited a much higher *k*_r_ value in contrast to DNS, demonstrating the importance of PPD in accelerating the radiative decay from the excited state.

Increasing DNS conjugate loading from 0 to 80 mol% also caused a 99% reduction in PPD steady-state fluorescence anisotropy (Fig. S12), which is a measure of fluorophore orientation and rotation receptive to excitation by plane-polarized light. The ultrafast anisotropy decay of PPD (80 mol% loading ratio of DNS) suggested that the excitation energy can readily delocalize over the whole PPD. In consequence, the excitation energy in a DNS that absorbs a photon will efficiently migrate to neighboring dyes because the DNS molecules are closely coupled within PPD and share compatible absorption transition moments (ATMs).

**Fig. 2:**
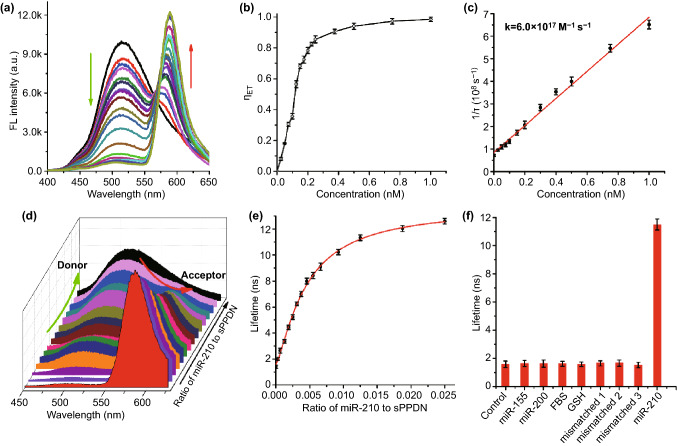
**a** Fluorescence spectra of sPPDN (DNS: 1 µM) in aqueous solution with different concentrations of SCO-Rb. The concentrations of SCO-Rb were 0.00, 0.01, 0.025, 5, 0.075, 0.1, 0.125, 0.15, 0.175, 0.2, 0.225, 0.25, 0.5, 0.75, 1 nM. **b** Energy transfer efficiency as a function of SCO-Rb concentration in sPPDN. **c** Plots of the reciprocal of the lifetime of the PPDN containing different concentrations of Rb against the concentrations of the acceptors. The slope gives the corresponding second-order rate constant (k) for the excitation energy migration toward the acceptor. Response of sPPDN to the nucleic acid target. **d** Fluorescence spectra of sPPDN after incubation with target miR-210 with different concentrations. **e** Fluorescence lifetime of DNS after incubation with the target at different concentrations of miR-210. **f** Fluorescence lifetime change of sPPDN for miR-210, miR-200, miR-155, FBS, GSH, and the mismatched sequences of miR-210

We selected Rb as the FRET energy acceptor for the sPPDN light-harvesting system because the fluorescence excitation spectrum of Rb overlaps well with the emission spectrum of DNS (Figs. 1f and S13-S14). Using steady-state and time-resolved fluorescence spectroscopy, we demonstrated that gradually increasing SCO-Rb concentration in vitro resulted in simultaneous acceptor emission (λ_em_ = 580) intensity elevation and donor emission (λ_em_ = 510) intensity depression for the sPPDN assemblies (Fig. 2a). This effect depended upon the hybridization of SCO-Rb conjugates to the ssDNA-210-DBCO probe. Subjecting SCO-Rb to PPD that is unconjugated to ssDNA-210-DBCO resulted in weak interaction between SCO-Rb and PPD and therefore minimal fluorescence (Fig. S15). Evaluating the fluorescence of sPPDN comprised of the different donor (D) to acceptor (A) fluorophore molar ratios revealed that when the D/A molar ratio reached 1000/1, or 1 µM DNS to 1 nM SCO-Rb in solution, DNS emission became nearly 99% quenched (Fig. 2b). The addition of Rb dyes to the nanoantenna also corresponds to a bathochromic (red) shift in the emission spectrum. Assessing DNS fluorescence lifetime over the same range revealed a large drop in the exponential decay of fluorescence when the D/A molar ratio reached 1000/1, with DNS lifetime curbing from an initial value of τ = 12.4 ns to τ = 1.4 ns (Fig. S16 and Table S4). Adjusting the D/A molar ratio in sPPDN also permits subtle tuning of the sPPDN’s fluorescence emission color. In Fig. S17, the emission spectrum of sPPDN modified with gradually increasing D/A molar ratios is overlayed on a 2D projection of the CIE (Commission Internationale de l’Eclairage) chromaticity diagram. The projection illustrates a smooth color transition from green to red as the SCO-Rb concentration increases, revealing both efficient FRET performance and a spectral dimension suitable for evaluating the abundance of molecular targets based on changes in fluorescence decay rates (Fig. S17). We calculated the second-order reaction constant of excitation energy transfer from D to A to be k = 6.0 × 10^–17^ M^−1^ s^−1^ (Fig. 2c). The capacity for sPPDN assemblies to perform light harvesting is derived from an energy-channeling operation called the antenna effect, which is an empirical parameter that quantifies the degree of amplified emission resulting from excitation of an energy donating fluorophore. In the sPPDN system, the maximum acceptor emission amplification factor was 70.5 at a D/A molar ratio of 2000/1 (Fig. S18). A signal amplification factor of this magnitude is imperative for low abundance miRNAs to be detected and quantified effectively. Using the estimated PEG-DNS conjugate loading within PPDN of 80 mol% and 38.4 nm as the average diameter of PPDN assemblies, we projected that each PPDN should contain 4250 DNS dyes. Remarkably, only two ssDNA-210: SCO-Rb hybrids are required for FRET to proceed from D to A at 94% efficiency. Consequently, disrupting the interactions between two Rb-linked competitive nucleotide sequences hybridized to the nanoantenna will reduce the fluorescence lifetime of DNS. The resulting shift in lifetime detected during FRET experiments may be mapped using fluorescence lifetime imaging microscopy (FLIM), which offers higher spectral sensitivity than intensity-based FRET.

The sPPDN assemblies were then incubated with various concentrations of miR-210 (complementary target) at 37 °C for 30 min. In the presence of the miR-210, fluorescence intensity at 580 nm decreases and the one at 510 nm increases, which is relative to miR-210 concentration (Fig. 2d). The obtained sPPDN showed a strong ratiometric response to the target miR-210 sequences, in which the target sequence hybridized with the ssDNA-210 sequence at sPPDN surface, thus stopping FRET (Fig. S19). Meanwhile, the time-resolved fluorescence measurements decay curves showed that the fluorescence lifetime of DNS was proportional to the ratio of miR-210 to sPPDN in the range from 0 to 0.025 (Fig. 2e).

To demonstrate the analytical specificity of the sPPDN, the sPPDN was incubated with the three mismatched sequences of miR-210 (100 pM), miR-155 (100 pM), and miR-200 (100 pM). The addition of miR-210 led to a significant increase in fluorescence lifetime, while no obvious fluorescence lifetime increase on others (Fig. 2f). BSA and glutathione (GSH) were also included with sPPDN and the lifetime changes were negligible for both, suggesting that the empirical capacity for sPPDN to interact compatibly for miR-210 detection with negligible signal interference within a biological environment.

### Light-Triggered Lysosomal Escape of PPDN

Besides its role as a fluorophore, Rb acts as a photosensitizer by catalyzing ROS formation in the presence of excitation light owing to its delocalized π systems. While SCO-Rb is susceptible to radiative shielding within sPPDN as it interacts with the ssDNA probe, measurements of ROS production using the singlet oxygen sensor green (SOSG) indicator suggested that SCO-Rb associated with sPPDN has a comparable rate of ROS generation to free Rb (Fig. S20).

We then monitored intracellular PPDN trafficking through colocalization experiments to establish the nanoparticle’s internalization pathway (see supporting information). Cellular uptake of PPDN nanoparticle assemblies diminished significantly in H1299 human lung cancer cells when incubated at 4 °C or in the presence of the ATPase inhibitor NaN_3_, indicating that endocytic loading of PPDN is an energy-dependent process (Fig. S21). Pretreating cells with dynasore, an inhibitor of dynamin-mediated vesicle scission, and chlorpromazine, which prevents assembly of clatherin-coated pits, resulted in significant reductions in sPPDN uptake efficiency, whereas treatment with neither the macropinocytosis inhibitor EIPA nor the caveolae-dependent endocytosis inhibitor filipin diminished uptake efficiency (Fig. S22). Thus, endocytosis by clatherin-coated vesicles is the dominant mechanism of PPDN internalization. FNPs incorporated into cells by clatherin-dependent endocytosis have been shown previously to ultimately resettle in lysosomes [42]. To interrogate the patterns of intracellular PPDN compartmentalization, we costained H1299 cells with CellLight Early Endosomes-RFP, CellLight Late Endosomes-RFP, and LysoTracker Red (Invitrogen, Carlsbad, CA). The fluorescent signal of PPDN closely overlaps with early-stage endosomes that recently internalized the PPDN (Fig. S23), but the wide distribution of PPDN beyond the area of late-stage endosomes suggests that the PPDNs are conveyed to different compartments or escape the endomembrane system altogether (Fig. S24). Spatial overlap of PPDN with LysoTracker Red-stained lysosomes observed in 3D demonstrates that most PPDN eventually relocated to lysosomes (Fig. S25).

**Fig. 3:**
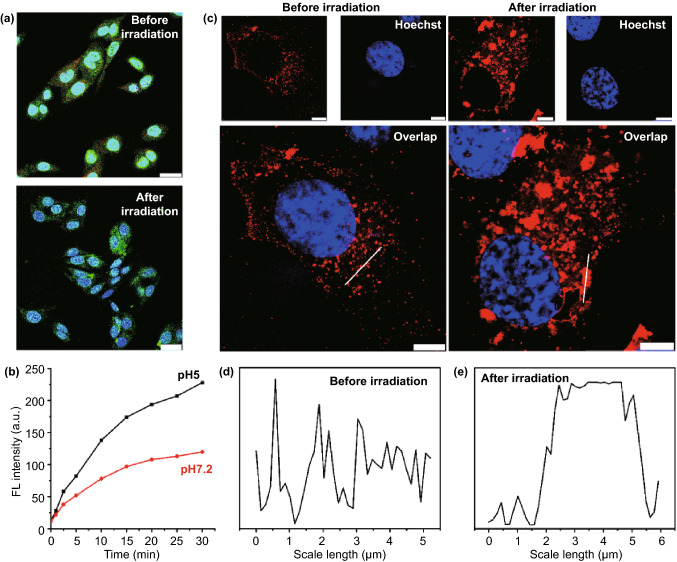
Lysosomal escape of sPPDN. **a** Observation of lysosomal disruption of H1299 cancer cells treated with sPPDN before and after light irradiation (10 mW cm^−2^, 5 min) using AO staining. AO generates red fluorescence in acidic lysosomes and emits green fluorescence in cytosol and nucleus. Scale bar = 20 μm. **b** Fluorescence intensity of sPPDN in H1299 cancer cells under different pH conditions. **c** Confocal fluorescence images of H1299 cancer cells stained by Hoechst 33,342 after incubation with sPPDN (250 nM) for 2 h before and after light irradiation (10 mW cm^−2^, 5 min). Scale bars = 8 μm. **d** Corresponding fluorescence intensity profiles of the areas marked by white lines in **c** before irradiation. **e** Corresponding fluorescence intensity profiles of the areas marked by white lines in **c** after irradiation

Passage of nanoparticle assemblies into the cytosol requires disruption of the lysosomal membrane. ROS promote this effect by reacting with unsaturated fatty acids present in membrane phospholipids, resulting in lipid peroxidation and endomembrane penetration. During light irradiation, sPPDN yields ROS as a by-product, facilitating temporary disruption of lysosomal membranes. Acridine orange (AO) staining rendered non-permeabilized lysosomes red due to the relative acidity which characterizes lysosomes (Fig. 3a). In contrast, red fluorescence was not detected after 5 min of irradiation at 10 mW cm^−2^, indicating successful lysosomal permeabilization. A low intravesicular pH likely augments ROS production and thus lysosomal escape, as evidenced by greater gains in fluorescence intensity over time when sPPDN were incubated in NaAc buffer (pH 5) compared to PBS buffer (pH 7.2) (Fig. 3b). Cells treated with sPPDN receiving the same irradiation treatment exhibited vacuolated structures that are likely swollen lysosomal aggregates agitated by ROS-induced oxidative stress (Fig. 3c). Sampling fluorescence intensity along arbitrary linear segments of cytoplasm corroborates this premise (Fig. 3d, e). Altogether, these results demonstrate a clear pathway for sPPDN escape from lysosomes after cellular uptake. Moreover, the photostable study of our probe in cell culture medium was investigated. The nanoprobe was continuously irradiated for different time intervals. Then, the fluorescence photobleaching curves of each sample were obtained by a spectrofluorimeter (λ_em_ = 580 nm). As shown in Fig. S26, after 10 min, the fluorescence emission intensity of the nanoprobe remained nearly unchanged, indicating good photostability in the cell culture medium.

### Cytosolic Detection of miRNA by sPPDN

We performed colocalization experiments using confocal microscopy to confirm that sPPDN are capable of miRNA biosensing within the cytosol after irradiation (Figs. S27-S28). As shown in Fig. S27, H1299 cells first exhibited bright red and dim green fluorescence signals with the sPPDN after 2 h incubation. After light irradiation, Rb fluorescence decreased and DNS fluorescence increased. After incubation for another 2 h, the Rb fluorescence intensity decreased to the minimal value and the semiquantitative FRET efficiency parameter, A/(A + D), decreased from 0.92 down to 0.25 (Fig. S28). The longer incubation times can increase the sensitivity, which might be attibuted to the sufficient diffusion process of nanoprobes and the hybridization reaction kinetics.

**Fig. 4:**
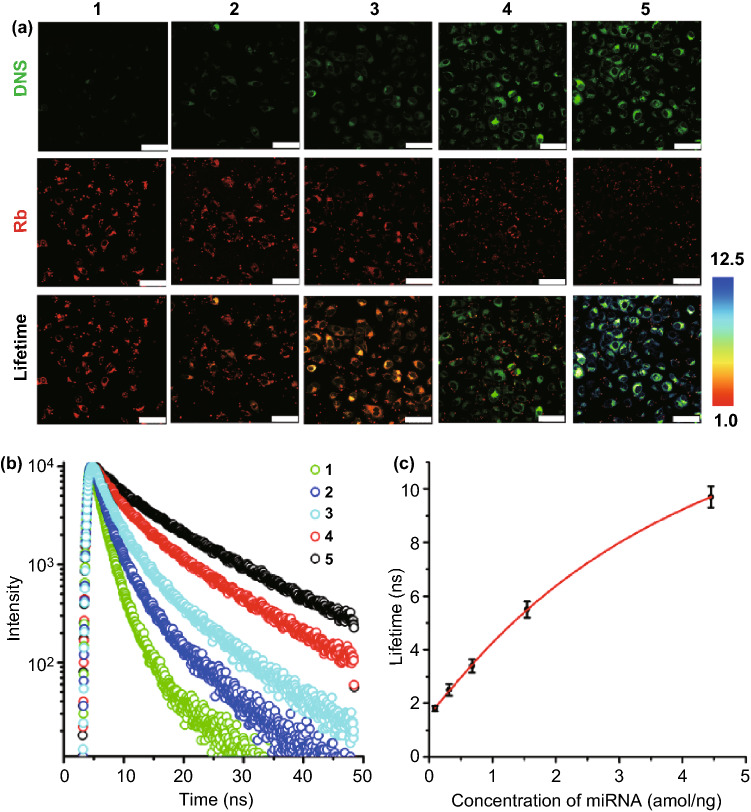
sPPDN for miR-210 detection in living cells. **a** Confocal images and fluorescence lifetime images of H1299 cancer cells with different amounts of miR-210: Columns 1 and 2 were transfected with antisense sequences of miR-210, column 3 was not transfected, and columns 4 and 5 were transfected with both miR-210. Scale bars: 50 μm. **b** Relative fluorescence lifetime decay curves of DNS in H1299 cancer cells with different amounts of miR-210. **c** The lifetime for different concentrations of intracellular miR-210. The excitation wavelength was light irradiation (10 mW cm^−2^, 5 min). Signals from the donor and the acceptor were recorded at 510 and 580 nm, respectively

FLIM-FRET measurements of miRNA-210 H1299 cancer cells subjected to photobleaching are presented in Figs. S29-S31 to display the effects on lifetime. Next, we transfected H1299 cancer cells with different amounts of miR-210 or antisense miR-210 to create cell lines expressing controlled levels of miR-210 for experimental quantitation (Figs. 4 and S32-S33). Each of the five cell lines expressing different levels of cytosolic miRNA was verified by real-time RT-PCR. Both fluorescence emission intensity and fluorescence lifetime of DNS increased as the intracellular miR-210 concentration increased (Fig. 4a). We observed that the H1299 cells transfected with antisense miR-210 exhibited relatively short lifetimes of 1.8 and 2.5 ns, whereas the H1299 cells transfected with miR-210 exhibited relatively long lifetimes of 5.5 and 9.7 ns (Fig. 4b). The lifetime of DNS is linearly related to the concentration of intercellular miR-210 in the range of 0.032–2.97 amol/ngRNA (Fig. 4c) with adjusted R^2^ = 0.99. We calculated the LOD as 3α/*slope*, where α represents the standard deviation of the negative control and *slope* is obtained from the linear calibration plot [43]. The LOD was 8.3 zmol/ngRNA (0.29 amol). To the best of our knowledge, this method represents the most sensitive detection protocol for intracellular miR-210 reported to date.

**Fig. 5:**
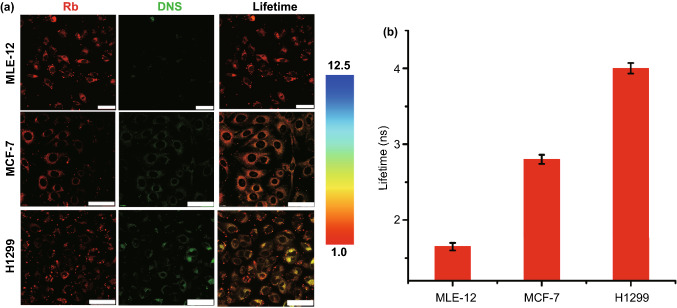
**a** Confocal images and fluorescence lifetime images for MLE-12, MCF-7, and H1299 cells with sPPDN. Scale bar: 50 µm. **b** Summarized data of lifetime in different cell lines toward sPPDN treatment. Error bars are mean ± SD (n = 3 independent samples)

To test whether sPPDN is compatible as a miRNA biosensor in other cell lines, we incubated MLE-12 cells and MCF-7 cells with sPPDN alongside H1299 cells and then estimated miR-210 levels using the procedure described above. As shown in Fig. 5a, the expression of miR-210 in MLE-12 cells was low as indicated by the faint DNS signal, indicating high FRET performance and a short fluorescence lifetime. The emission intensity of DNS was relatively high in H1299 cells compared to MCF-7 cells, but Rb had relatively weak fluorescence, as expected, due to SCO-Rb decoupling from the sPPDNs. FLIM measurements indicated that the MLE-12 and MCF-7 cells exhibited lifetimes of 1.65 ± 0.1 and 2.8 ± 0.13 ns, respectively, but H1299 cells had a relatively long lifetime of 4 ± 0.2 ns, suggesting that the level of miR-210 in those cells was higher than in MCF-7 cells (Fig. 5b). According to the calibration curves, we found miR-210 levels to be 0.06 amol/ngRNA in MLE-12 cells (or 2.3 amol miR-210), 0.44 amol/ngRNA in MCF-7 cells (or 15.3 amol miR-210), and 0.98 amol/ngRNA in H1299 cells (or 34.1 amol miR-210), respectively. These results are consistent with the miR-210 concentrations obtained from RT-PCR data (0.068 amol/ngRNA in MLE-12 cells, 0.48 amol/ngRNA in MCF-7 cells, 0.92 amol/ngRNA in H1299 cells) and previously reported values for miRNA (Fig. S34) [44].

Once the feasibility of miR-210 detection with sPPDNs in living cell lines was confirmed, the same sPPDNs were tested in deep tissues of mice bearing tumor xenografts. In a typical experiment, tumor xenografts were generated by the s.c. injection of female nude mice with H1299 cells. Upon tumor formation, the sPPDNs were injected into the mice through their tail veins. 

**Fig. 6:**
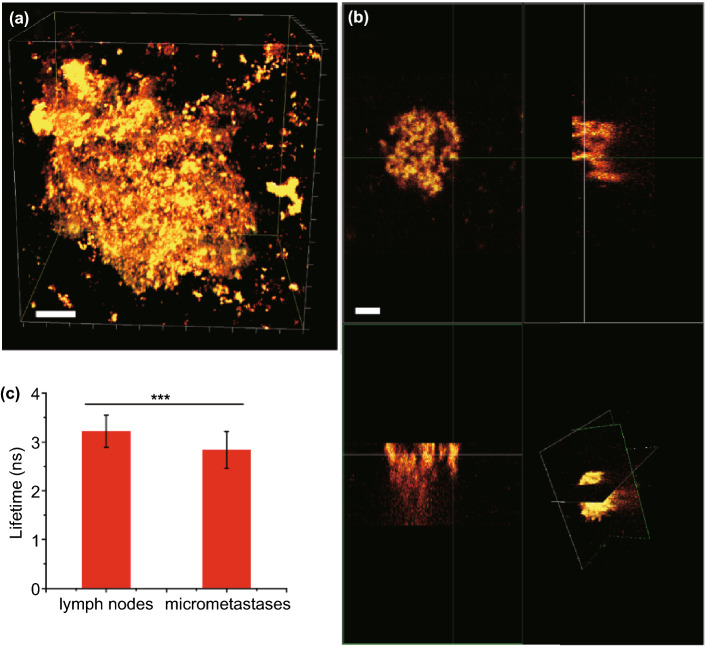
**a** In vivo images showing the FLIM images of in H1299 tumor cells in agarose sections of lymph nodes from in vivo mouse models**.** Scale bar: 200 µm. **b** 3D FLIM image of a micrometastasis. Scale bar: 100 µm. **c** Relative lifetime values of mice in lymph nodes and micrometastasis. 10 independent optical slices were randomly selected. Means of sets of data were statistically evaluated using a two-tailed unpaired t test on GraphPad Prism (confidence interval: 95%). ***P < 0.01 (Welch’s t test)

 We used agarose slices of lymph nodes infiltrated by tumor cells from the H1299 tumor mouse models. FLIM measurements indicated that the lymph nodes exhibited an average lifetime of 3.2 ± 0.3 ns from the tumor cell, which found the miR-210 levels to be 0.58 amol/ngRNA (Fig. 6a). The capacity of sPPDNs to specifically detect miR-210 in tumor cells was not limited to lymph node metastases, but was also shown for lung micrometastases (Fig. 6c). The difference between these groups is statistically significant (***P < 0.01). As shown in Fig. 6b, 3D section of FLIM measurements indicated that the lung micrometastases exhibited the average lifetime of 2.8 ± 0.4 ns from the tumor cell, which found the miR-210 levels to be 0.44 amol/ngRNA, demonstrating that sPPDNs have a high potential as imaging probes for the detection of miR-210 in lung micrometastases tissue sections.

## Conclusions

We have designed a light-harvesting nanoantenna and achieved the quantification of low-abundance cytosolic miRNAs with high precision in vitro and in vivo imaging. Nanoantennae containing over 1000 donor dyes can efficiently transfer excitation energy to a single FRET acceptor hybridized at the ssDNA probe-modified surface with 99% efficiency, leading to exceptional signal amplification and biosensing sensitivity. Dense packing of DNS dyes (80 mol%) in this nanoantenna ensures short inter-fluorophore distances with minimal ACQ which facilitates efficient excitation energy migration between the encapsulated DNS molecules. The nanoantenna’s high energy transfer efficiency also enhances ROS generation upon irradiation. In doing so, the lysosomal membrane ruptures due to oxidative stress, thereby unloading the nano-antennas into the cytosol. The sPPDN can be used to quantify cytosolic miR-210 at the zeptomolar scale with high accuracy and precision. It may be a suitable tool for cancer diagnosis and bioimaging in mice and may inspire further exploration of nano-light-harvesting systems.

## Supplementary Information

Below is the link to the electronic supplementary material.

Supplementary file1 (PDF 1800 kb)
