# Supplementary material for: Zeptomole Imaging of Cytosolic MicroRNA Cancer Biomarkers with A Light-Controlled Nanoantenna
Source: Nanomicro Lett. 2021 Oct 21;13:213. doi: 10.1007/s40820-021-00732-1 (PMC8531139; doi:10.1007/s40820-021-00732-1)
Supplement: Supplementary file 1 — Supplementary file1 (PDF 1800 kb) [file 40820_2021_732_MOESM1_ESM.pdf]

Supporting Information for

Zeptomole Imaging of Cytosolic MicroRNA Cancer Biomarkers with A Light-Controlled Nanoantenna

Yang Song[1, 2, #], Xiaoli Cai[1, #], Grayson Ostermeyer[3], Shichao Ding[1], Dan Du[1], Yuehe Lin[1, *]

[1]School of Mechanical and Materials Engineering, Washington State University, Pullman, Washington 99164, United States

[2]Nanosong Systems LLC, Redmond, Washington 98052, United States

[3]School of Biological Sciences, Washington State University, Pullman, Washington 99164, United States

[#]Yang Song and Xiaoli Cai contributed equally to this work

*Corresponding author. E-mail: yuehe.lin@wsu.edu (Yuehe Lin)

S1 Experiment

S1.1 Materials

Methoxy PEG550 Succinimidyl Succinate (mPEG-SS), Folate PEG550 Succinimidyl Succinate (fPEG-SS) and Azide PEG550 Succinimidyl Succinate (aPEG-SS) were purchased from Jenkem Technology (TX, USA). AminopropylIsobutyl POSS was purchased from Hybrid Plastics Inc (MS, USA). 5-(Dimethylamino)-1-naphthalenesulfonamide (99%) (DNS), Ascorbic acid (AA), N,N-dimethylformamide (anhydrous, 98%), N,N-Diisopropylethylamine (≥99%), acetonitrile (anhydrous, 99.8%), dichloromethane (anhydrous, ≥99.8%), 1-Hydroxybenzotriazole (≥97%), singlet oxygen sensor green (SOSG) indicator, diamidino-2-phenylindole (DAPI), deoxyribonucleic acid sodium salt from calf thymus (Type I, fibers), albumin bovine serum (Fraction V, minimum 96%, lyophilized powder), Amicon Centrifugal filters (0.5mL, 100K) were purchased from Sigma-Aldrich. Citric acid monohydrate (≥99.5%), sodium azide (99%), sodium iodide (≥99.5%) and trifluoroacetic acid (99%) were purchased from Alfa Aesar. Sulfo-Cyanine3 azide (95%) was purchased from Lumiprobe. CellLight Early® Endosomes-RFP, CellLight Early® Late Endosomes-RFP, LysoTracker® Red DND-26, CellROX™ Deep Red Reagent, F12K medium (Gibco®) and FBS (Gibco®) were purchased from ThermoFisher Scientific. H1299 cells (CRL-5803), penicillin and streptomycin were purchased from American Type CultureCollection (ATCC). Sodium phosphate monobasic (>99.0%, Sigma-Aldrich) and sodium phosphate dibasic dihydrate (>99.0%, Sigma-Aldrich) were used to prepare 1× PBS at pH 7.4. For saline buffer sodium chloride (≥99%, Sigma Aldrich) 30 mM and magnesium chloride (≥98%, Sigma Aldrich) 12 mM was added to 20 mM phosphate buffer and pH was adjusted with sodium hydroxide solution. Millipore ultrapure water was used throughout the experiments. All the reagents and solvents were commercially available and used as received unless otherwise specified purification.

S1.2 Characterization

UV-vis spectra were measured by UV-2450 spectrometer in a quartz cell (light path 10 mm) at 25 °C. The Steadystate fluorescence spectra were measured in a conventional quartz cell (10 × 10 × 45 mm) at 25 °C by a Cary Eclipse fluorimeter. The fluorescence lifetimes were measured

by timecorrelated single photon counting on a FLS920 instrument (Edinburg Instruments Ltd., Livingstone, UK) with a H_2 pulse lamp. Transmission electron microscopy (TEM) samples were prepared by pipetting one drop of water diluted peptoid suspensions onto carbon-coated electron microscopy grid; 2% phosphotungstic acid was then used for negative staining. TEM measurements were conducted on an accelerating voltage of 200 kV FEI Tecnai TEM microscope. Dynamic Light Scattering (DLS) measurements were recorded by Nano ZS equipped with a solid-state He-Ne laser (λ=633 nm). Confocal imaging was performed with a confocal laser fluorescence microscope (Leica SP8 Point Scanning Confocal).

S1.3 Synthesis of MeO-PEG550-POSS and MeO-PEG550-POSS-DNS

POSS was first conjugated with mPEG-SS, fPEG-SS and aPEG-SS, then mPEG-POSS was then conjugated with DNS. Brefilly, POSS (10 mg) was dissolved in 1 mL of DMSO and mixed with 60 µL of TEA to neutralize the ammonium site of the POSS. Then 100 mg of mPEG-POSS was disslved in 10 mL of DMSO and slowly injected to the POSS solution with strring. 20 mg of DNS was dissloved in DMSO and mixed with 25 mg of NHS and 25 mg of EDC to active the DNS molecules. After reaction for 2 h, the activated DNS was mixed with mPEG-POSS solution for 24 h at 4 ℃. Then the mixture solution was purfied using PD-10 Desalting Columns. The PEG-POSS-DNS power was obtained after lyophilization.

S1.4 HPLC Purification

Crude mPEG-POSS, fPEG-POSS, aPEG-POSS and PEG-POSS-DNS were dissolved in H_2O/CH_3CN (v/v=5:5) for HPLC purification (reverse-phase HPLC on a XBridgeTM Prep C18 10 µm OBDTM, 10 µm, 19 × 100 mm^2). Purified mPEG-POSS, aPEG-POSS or PEG-POSS-DNS were analyzed using Waters ACQUITY reverse-phase UPLC (corresponding gradient at 0.4 mL min^{-1} over 7 min at 40 °C with an ACQUITY®BEH C18, 1.7 µm, 2.1 × 50 mm^2 column) that was connected with a Waters SQD2 mass spectrometry system. The final product was lyophilized to yield the pure powder (white powder for mPEG-POSS and aPEG-POSS, and light-green yellow powder for PEG-POSS-DNS, respectively).

S1.5 Estimation of the Number of Dyes per PPD

To calculate nanoparticle concentration you must first determine the total mass of the element of interest in nanoparticle form in the solution. qNano was directly used for measuring nanoparticle concentration in solution. For a typical PPD solution, the number concentration (N) of the nanoparticles in units of PPD/L was measured by qNano. The particle molarity is calculated as the equation:

$$M = \frac{N}{\text{Avogadro constant}} \qquad \text{(S1)}$$

Based on loading of PEG-POSS-DNS/mPEG-POSS/fPEG-POSS/aPEG-POSS polymer mixture (optimized molar ratio: 80:18:1:1) in PPD, we calculated the number of moles of the POSS molecule in the typical PPD solution. The latter value of the estimated number of POSS molecules per particle (~2951) was calculated by the equation:

$$N_{each} = \frac{N_{poss}}{\text{M×V}} \qquad \text{(S2)}$$

where N_{each} represents the number of POSS molecules per particle, N_{poss} is the number of moles of the POSS molecule in the typical PPD solution, M is the particle molarity and V is the volume of PPDN solution.

Based on loading of the PEG-POSS-DNS into the PPD (80 mol%), we calculated the number of moles of the PEG-POSS-DNS of the single particle. The latter value was multiplied by the

DNS/POSS molar ratio (1.8) giving the estimated number of DNS dye molecules per particle (~4250).

S1.6 Energy-transfer Efficiency (Φ_{ET})

Energy-transfer efficiency, Φ_{ET}, the fraction of the absorbed energy that is transferred to the acceptor is experimentally measured as a ratio of the fluorescence intensities of the donor in the absence and presence of the acceptor (I_D and I_{DA}):

$$\Phi_{ET} = 1 - \frac{I_{DA}}{I_D} \qquad \text{(S3)}$$

The energy-transfer efficiency (Φ_{ET}) was calculated in water, measured in the condition of PPDN (10 μM) and Rb with different concentration, λ_{ex}=365 nm.

S1.7 Antenna Effect

The antenna effect under certain concentrations of donor and acceptor equals the ratio of the emission intensity at 580 nm of the acceptor upon excitation of the donor.

$$\mathrm{A} = \frac{I_{A+D_{365}}}{I_{A+D_{365}} - I_{A+D_{530}}} \qquad \text{(S4)}$$

where I_{A+D} (λ_{ex} = 365 nm) and I_{A+D} (λ_{ex} = 530 nm) are the fluorescence intensities of excitation of the donor at 365 nm and direct excitation of the acceptor at 530 nm, respectively. The antenna effect value was calculated as 70.5 in PBS solution, measured in the condition of PPDN (10 μM) and Rb (50 nM), λ_{ex}=365 nm.

S1.8 Exciton Migration Rate

The acceptor molecules are immobilized within the NPs surface, and only the excitons are migratory. Thus the quenching rate equation can be written:

$$\frac{d[D]}{dt} = -k[D^*][A] - k_0[D^*] \qquad \text{(S5)}$$

Where D is the donor and A refers to the acceptor. Since the excitation power is very low in the single-photon counting fluorescence lifetime measurement, we can assume that [A] >> [D*].

Thus:

$$[D^*] \propto e^{-(k'+k_0)t} = e^{\frac{1}{\tau}t} \qquad \text{(S6)}$$

where k equals the slope, the intercept is k_0 which is the reciprocal of the lifetime of the donor. For both acceptors, the rate constant is in an order of 10^{13} M^{-1} s^{-1}, which is apparently not only much larger than the diffusion limit for the bimolecular reaction in the solution. Therefore, the much larger second order rate can be ascribed to the favorable spatial orientation as well as a possible shorter mean average distance in contrast to those in the solution phase. Our result indicates that the excitonic energy mitigation rate within the nanocrystal would be much larger than that of the diffusion limit in the solution.

S1.9 Cell Culture

H1299 cancer cells were cultured in F-12K Medium (F-12K, GIBCO) with 10% fetal bovine serum (FBS, Invitrogen, Life Technologies) in a 5% CO_2 incubator at 37 °C. For imaging experiments, cells were seeded on a Petri dish (Corning) at a density of 5×10^5 per well and cultured overnight.

S1.10 Confocal Imaging

Confocal images were recorded with a Leica TCS SP8 confocal microscope equipped. The PPDN were excited with a 365 nm light, while the endosomes-RFP (CellLight Early® Endosomes-RFP and Late Endosomes-RFP, BacMam 2.0, Life Technologies) and LysoTracker Red dyes (LysoTracker® Red DND-26, Life Technologies) were excited with a 568 nm laser. Fluorescence images were analyzed using Fiji software (NIH).

Briefly, for endosome staining, H1299 cancer cells were cultured in 35 mm Petri dishes overnight. H1299 cancer cells were then incubated with staining solution (CellLight® Early Endosomes-RFP or Late Endosomes-RFP, BacMam 2.0, Life Technologies) overnight then incubated with PPDN for different time. The culturing supernatant of H1299 cells was removed and the cells were washed with 1 × PBS buffer (pH 7.4) three times. Finally, the cells were fixed with 4% paraformaldehyde (PFA) for 15 min and imaged using CLSM to observe co-localization of PPDN with endosomes.

For lysosome staining, cells were first incubated with PPDN for different time, then washed and incubated in a fresh medium containing 50 μM probes (LysoTracker® Red DND-26, Invitrogen) for 5 min. Finally, cells were washed with 1 × PBS buffer (pH 7.4) and fixed with 4% paraformaldehyde (PFA) for 15 min and imaged using CLSM.

The fluorescence ratio of donor (D, 510 nm) image to acceptor (A, 580 nm) image at different incubation conditions was calculated in the individual cell after exciting at the donor. The mean from the distribution of D to A ratio of the individual cell was obtained at different conditions and plotted to obtain a calibration curve. For ratio measurements, cells were imaged in three channels to yield four images, (1) donor channel by exciting at 365 nm and collecting at 510 nm (2) Acceptor channel by exciting at 365 nm and collecting at 580 nm (3) donor channel by exciting at 560 nm and collecting at 580 nm (4) Acceptor channel by exciting at 560 nm and collecting at 580 nm. Cross talk was measured with donor only and acceptor only samples and found to be negligible while around 15% of Rb was directly excited at 560 nm excitation and subtracted from the corresponding donor for representative images. Autofluorescence was measured on unlabelled cells. All the images were then background subtracted taking mean intensity of the cytoplasm, donor and acceptor images were co-localized andendosomes showing co-localization were analyzed using ImageJ software (NIH). Total intensity, as well as mean intensity in each endosome, was measured in donor and acceptor channels and a ratio of donor to acceptor intensities (D/A) of each endosome was obtained.

S1.11 Time-resolved Fluorescence Spectroscopy

Time-resolved fluorescence measurements were performed with the time-correlated, single photon counting technique using the frequency doubled output of a Ti-Sapphire laser (Tsunami, Spectra Physics), pumped by a Millenia X laser (Tsunami, Spectra Physics). The excitation wavelength was set at 350 nm. The fluorescence decays were collected at the magic angle (54.7º) of the emission polarizer. The single-photon events were detected with a microchannel plate Hamamatsu R3809U photomultiplier coupled to a Philips 6954 pulse preamplifier and recorded on a multichannel analyzer (Ortec 7100) calibrated at 25.5 ps/channel. The instrumental response function was recorded with a polished aluminum reflector, and its full width at half-maximum was 50 ps. Time-resolved decays were analyzed both by the iterative reconvolution method and the Maximum Entropy Method (MEM). The goodness of the fit was evaluated from the χ^2 values, the plots of the residuals and the autocorrelation function.

S1.12 Singlet Oxygen Detection in vitro

For extracellular detection, the singlet oxygen sensor green (SOSG) indicator was pre-mixed with the sample solution (free Rb in DMSO; sPPDN in PBS) at a concentration of 50 µM, then the sample solutions were exposed to laser irradiation at a power density of 10 mW cm^{-2} for 5 min. The emission of SOSG at 525 nm was recorded with a microplate reader at an interval of 2 min.

S1.13 Fluorescence miRNA Detection

For detection in solution, the sPPDN was diluted in 1 × PBS buffer containing to a desired concentration (10 µM) and aliquot of miRNA was added. Before measurements, the mixture was incubated in the dark at 4 °C. The mixture was kept in the dark at room temperature for different incubation time. Then, the fluorescence measurement was performed on the spectrofluorometer.

For miRNA detection in different biological media, the F12K medium was used for sample preparations. Fetal bovine serum (FBS) was diluted to the desired concentration.

S1.14 Calculating Intracellular miRNA210 in H1299 Cells

The synthetic miRNA210 were measured with the TaqMan® MicroRNA Assay and standard curves of the cycle threshold values (Ct) versus the concentrations of miRNA210 were constructed. The qRT-PCR amplification plots of the miRNA210 extracted from the regulated cells were constructed, and the Ct values for miRNA210 were then read from Fig. S29. The amounts of miRNA210 in the five samples were calculated from the standard. Transfection of Hela cells with miRNA210 and their antisense sequences. H1299 cancer cells, cultured for 12 h in culture plates with (initially) 1.0 × 10^6 cells, were transfected with different amounts of synthetic miRNA210, or antisense miRNA210. The transfection experiment was performed with Lipofectamine® RNAiMAX Transfection Reagent (Life Technologies), according to the manufacturer's instructions. Every cell medium was replaced with 10 mL of Opti-MEM containing RNAiMAX (300 µL) and the cells were transfected with miRNA210 (1, 10 pM) or antisense miRNA210 (1, 10 pM) for 24 h. After transfection, the intracellular miRNA210 concentrations were quantified using a commercially available miR-specific qRT-PCR kit.

S1.15 Quantification of miRNA210 in Transfected H1299 Cells

The miRNA210 in the transfected cells was extracted with the PureLink® RNA Mini Kit (Life Technologies) and quantified with the TaqMan® Small RNA Assay and ABI Prism® 7900HT22 PCR (Life Technologies).

S1.16 Confocal Imaging of Cytosol miRNA210 Detection

The transfected H1299 cells expressing different levels of miRNA210 were cultured on glass-bottomed Petri dishes for 12 h, at an initial density of 2 × 10^4 cells/dish. Then, the cells were incubated with the sPPDN. The sPPDN were prepared and incubated with the cells for 4 h at 37 °C. Then the cells were exposed to laser irradiation at a power density of 10 mW cm^{-2} for 5 min. The cells were washed and incubated in a fresh medium for another 4 h. Finally, cells were washed with 1 × PBS buffer (pH 7.4) and fixed with 4% paraformaldehyde (PFA) for 15 min and imaged using CLSM. Fluorescence lifetime images were obtained by using a Leica TCS SP8 confocal laser scanning microscope equipped with a TCSPC system. Different cells were imaged by an oil immersion objective (×63).

S1.17 Animal Tumor Models

Procedures involving animals and their care were conducted in conformity with institutional ethical guidelines and were approved by the Institutional Animal Care and Use Committee in a facility fully accredited by the Association for the Assessment and Accreditation of Laboratory Animal Care. Five-week-old female Balb/C nude mice were kept at 25 °C with humidity of 50%-60% and 12 h light-dark cycles. Mice were anesthetized using isoflurane (3%) carried with oxygen during injection. These nude mice were subcutaneously inoculated above the right flanks with 1.0 × 10^6 H1299 cells suspended in 50 μL of cold PBS into the right area to create breast solid cancer model. The *in vivo* studies were performed after 3 weeks. When tumors reached approximately 5 mm in diameter, the tumor-bearing mice were divided into 3 groups randomly. The 3 groups were treated respectively with different concentrations of miR-210 inhibitors: 0, 5, and 10 mg kg^{-1}, following different incubation time. For *in vivo* FLIM imaging, the mice were intravenously injected with sPPDN and incubated for another 12 h, the mice were irradiated at white light of 2.5 W cm^{-2} for 5 min. The GE eXplore Optix imaging system used for lifetime imaging study. H1299 tumor-bearing mice were imaged by positioning the mice on the animal plate, which was heated to 36 °C in the eXplore Optix system. Two-dimensional regions of interest were selected with the aid of a top real-time digital camera. The height of the animal was verified by a side digital camera to ensure the animal was in the correct plane of the imaging optics. The animal was automatically moved into the imaging chamber for scanning. Light power and count time settings were optimized at 0.95 W and 0.3 s per point. These parameters were kept constant throughout the imaging sessions. Excitation and emission spots were raster scanned in 0.5-mm steps over the selected region of interest to generate emission wavelength scans. The fluorescence emission was collected and detected through a fast photomultiplier tube (Hamamatsu, Japan) and a time-correlated single photon counting system (Becker and Hickl GmbH, Berlin, Germany). The data were recorded as temporal point-spread functions (TPSF) and the images were reconstructed lifetime maps.

S1.18 Delivery of miR-210 Inhibitors in vivo

The mirVanaTM miRNA Inhibitors were complexed with Invivofectamine 3.0 Reagent (Life Technologies) for *in vivo* applications. The miRNA oligonucleotides (2 or 3.5 mg mL^{-1} in 750 μL of water) were mixed with the manufacturer’s complexation buffer (750 μL), and then 1500 μL of Invivofectamine 3.0 Reagent was added. After incubation for 30 min at 50 °C, dialysis was performed against 4 L of PBS to remove excess salts and solvents. The resulting miRNA inhibitor concentration was 0.5 or 1.0 mg mL^{-1}. A 200 μL injection into the tail vein of each 20 g mouse, resulted in a final miRNA dose of 5 or 10 mg kg^{-1} body weight.

S1.19 Histology Imaging

Briefly, animals were deeply sedated and fixed via transcardial perfusion with monomer solution containing 2% wt/vol acrylamide, 4% formaldehyde, and 0.25% wt/vol VA-044 axoinitiator. The sentinel (axillary, brachial and popliteal) lymph nodes or organs were collected for histological analysis. The isolated fresh lymph nodes were directly placed into the frozen section compound (VWR®International, LLC95057-838) in a plastic cryomold (Tissue-Tek® at VWR®4565) and frozen with liquid nitrogen. The frozen samples were then gelled by removing air and purging with argon gas before incubating at 37 °C for 3 h. 1 μm tissue sections were cut on Cryostar NX70 and placed on charged slides. The tissue sections were scanned and imaging using an eXplore Optix system for all bright-field, fluorescent and FLIM images.

S1.20 Statistical Analysis and Reproducibility

The uptake of probes in cells was analysed by the background subtracted cells using ImageJ software (NIH), the colocalization was quantified using Colocalization finder plugin and the image quantification was done either in Origin software or using Prism. Mean values with associated standard deviation (SD) were measured at ten independent experiments and p values were calculated using two-tailed unpaired t-test on GraphPad Prism (confidence interval: 95%).

S2 Supplementary Figures and Tables

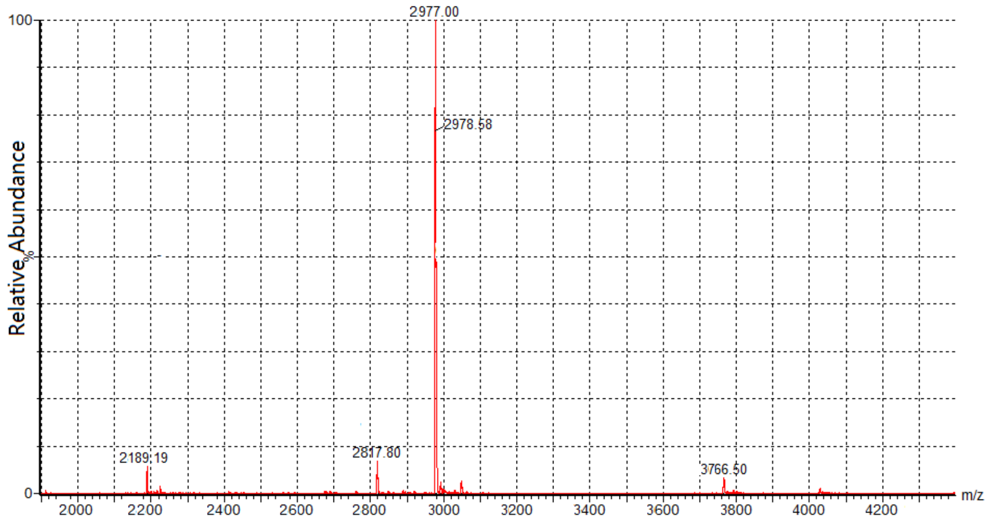

Fig. S1 The mass spectrum of mPEG-POSS

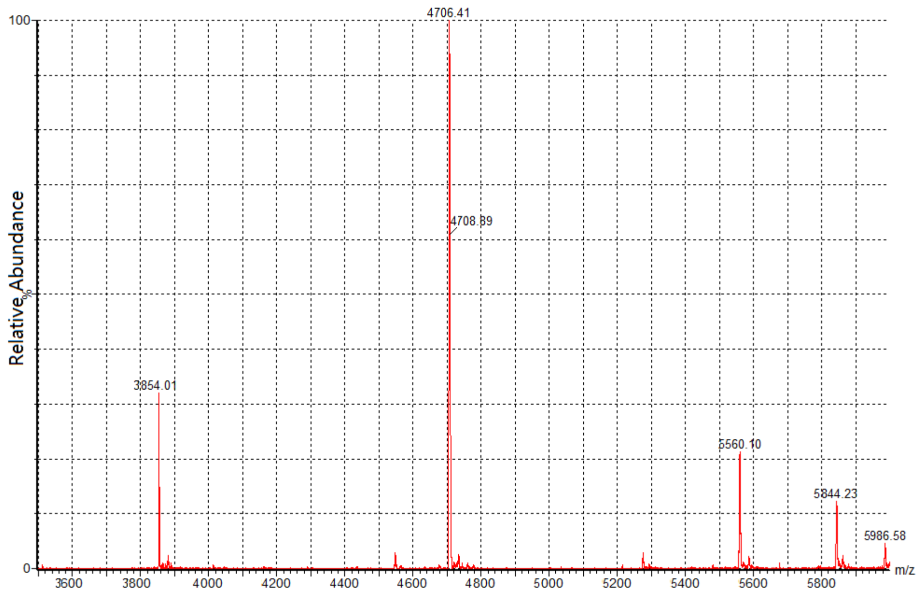

Fig. S2 The mass spectrum of PEG-POSS-DNS

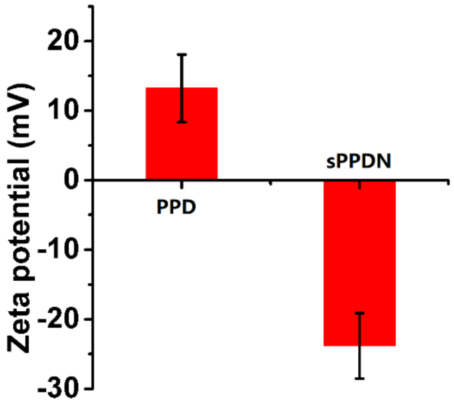

Fig. S3 Change in the zeta potential of PPD (10 μM) and sPPDN (10 μM) at 37°C in pH 7.4 buffer

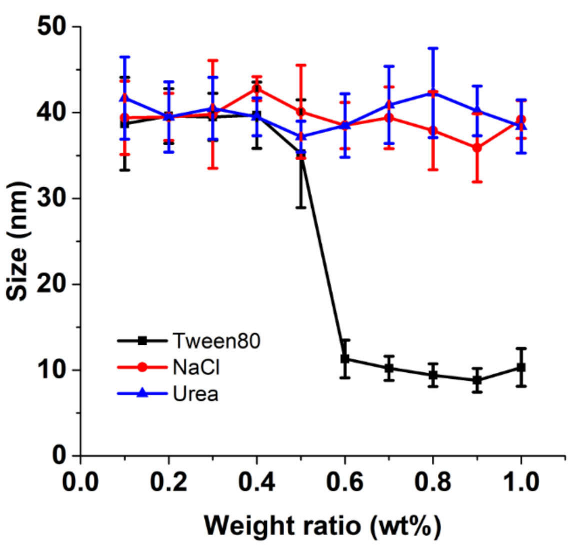

Fig. S4 The size changes of PPD after treatment with different concentrations of Tween-80, NaCl and Urea, respectively

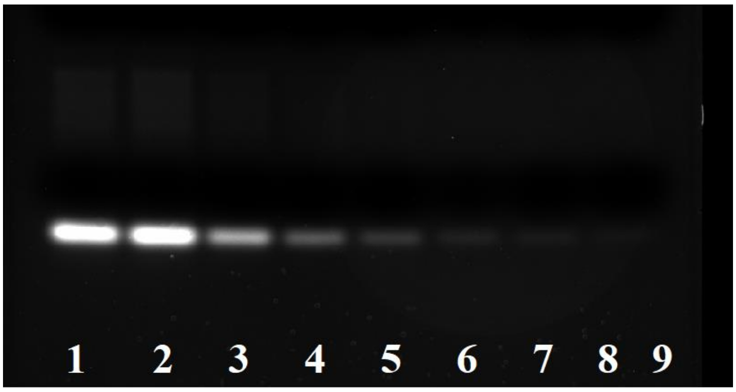

Fig. S5 Gel-retardation assay of PPD/ssDNA-210-DBCO at different ratios. Lane 1: ssDNA-210-DBCO only (3 μM); Lane 2: ssDNA-210-DBCO treated with PPD without azide; line 3: ssDNA-210-DBCO treated with PPD of 1 μM; line 4: ssDNA-210-DBCO treated with PPD of 2 μM; line 5: ssDNA-210-DBCO treated with PPD of 3 μM; line 6: ssDNA-210-DBCO treated with PPD of 4 μM; line 7: ssDNA-210-DBCO treated with PPD of 5 μM; line 8: ssDNA-210-DBCO treated with PPD of 7.5 μM; line 9: ssDNA-210-DBCO treated with PPD of 10 μM

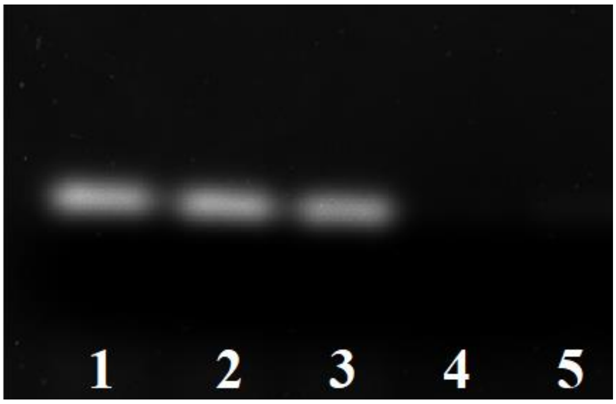

Fig. S6 Protection from DNAse digestion as evaluated by electrophoresis. Lane1: sPPDN only (1 µM); Lanes 2 and 3: sPPDN treated with DNAse for 20 (lane 2) and 60 (lane 3) min; Lane 4: ssDNA-210-DBCO only treated with DNAse for 20 min; Lane 5: ssDNA-210-DBCO treated with DNAse for 60 min

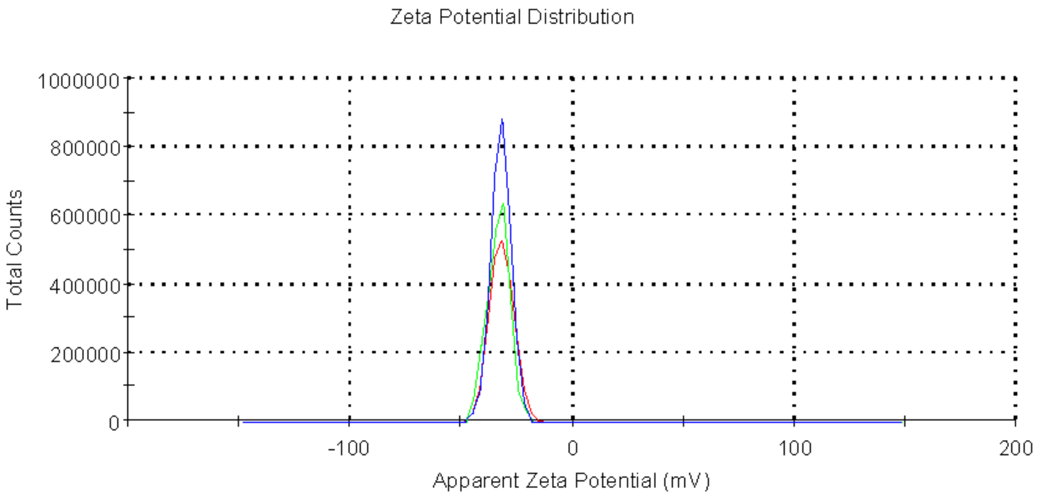

Fig. S7 The zeta potential changes of sPPDN after treatment with control (blue), 10% FBS (red) and human serum (green)

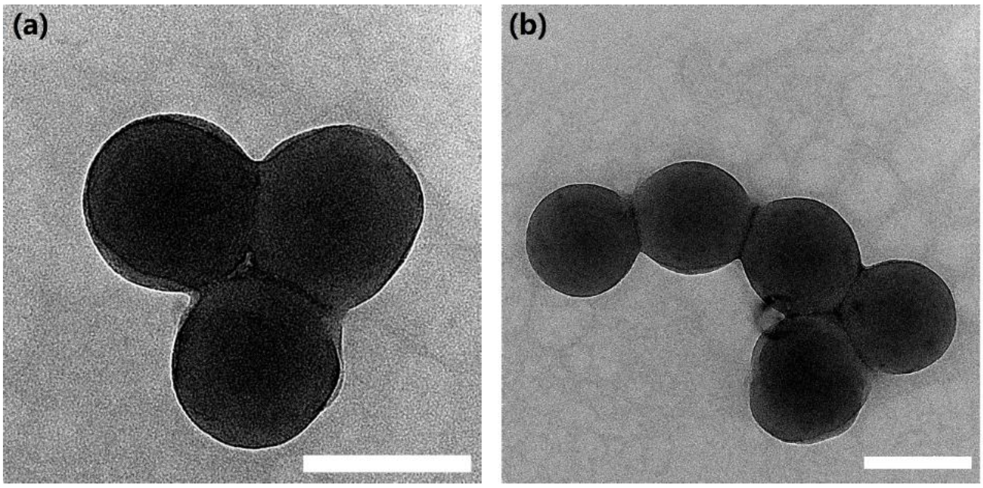

Fig. S8 (**a**) TEM image of sPPDN in borate buffer (pH 9.0); (**b**) TEM image of sPPDN in cell culture medium (10% FBS). Scale bar: 50 nm

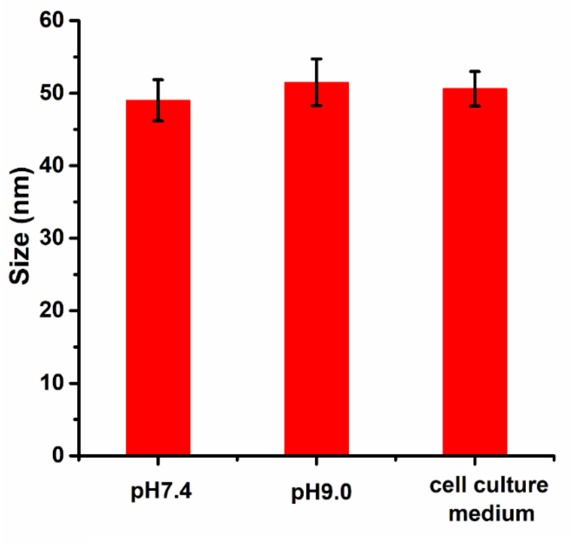

Fig. S9 The size changes of sPPDN in different buffer conditions: 1x PBS buffer (pH 7.4), 10 mM borate buffer (pH 9.0) and cell culture medium (10% FBS)

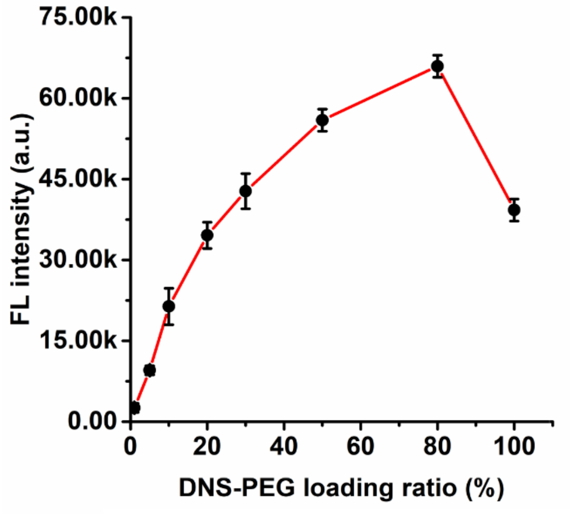

Fig. S10 Fluorescence intensity of PPD as a function of PEG-DNS loading ratio. Error bars represent standard deviation (n = 3)

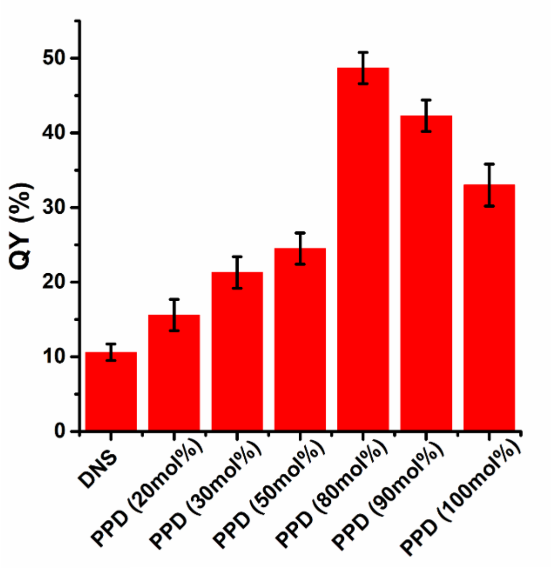

Fig. S11 Quantum yields of six different PPD and DNS dye in an aqueous solution

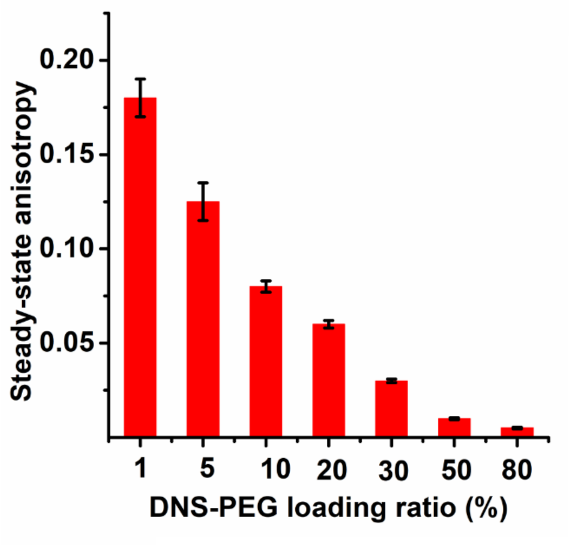

Fig. S12 Steady-state fluorescence anisotropy of PPD as a function of PEG-DNS loading. Error bars represent standard deviation (n = 3)

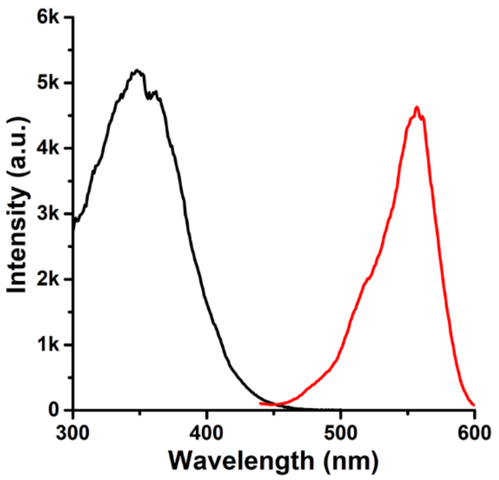

Fig. S13 Fluorescence excitation spectra of PPDN (black) and excitation spectra of Rb (red)

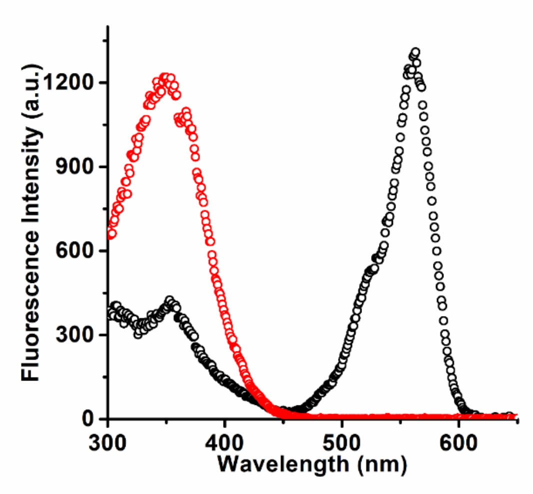

Fig. S14 Fluorescence excitation spectra of PPDN without (red) and with 50 nM of SCO-Rb (black)

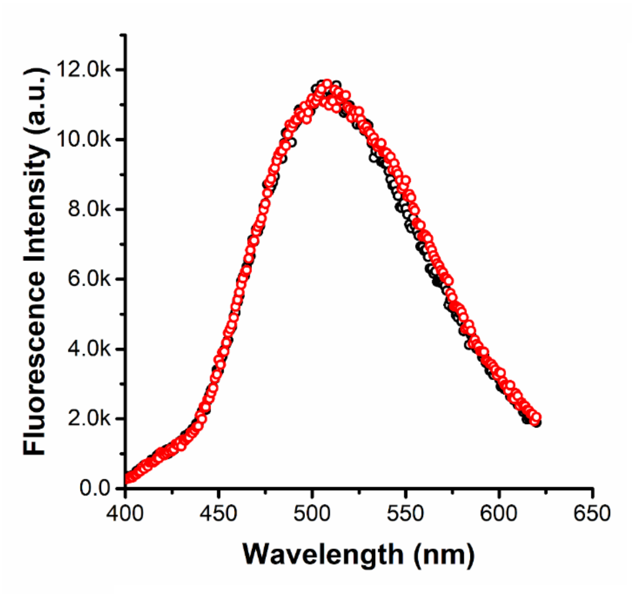

Fig. S15 Fluorescence spectra of PPD without ssDNA-210-DBCO sequence in water before and after addition of SCO-Rb (100 nM) (λ_{ex}=365 nm)

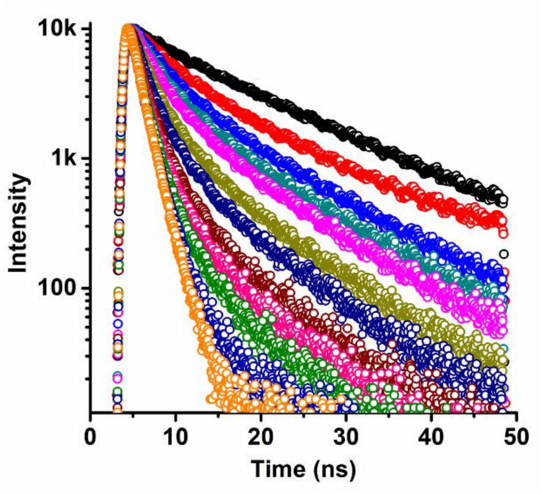

Fig. S16 Fluorescence lifetime decay curves of DNS of sPPDN in aqueous solution with different concentrations of SCO-Rb (excitation wavelength is 365 nm)

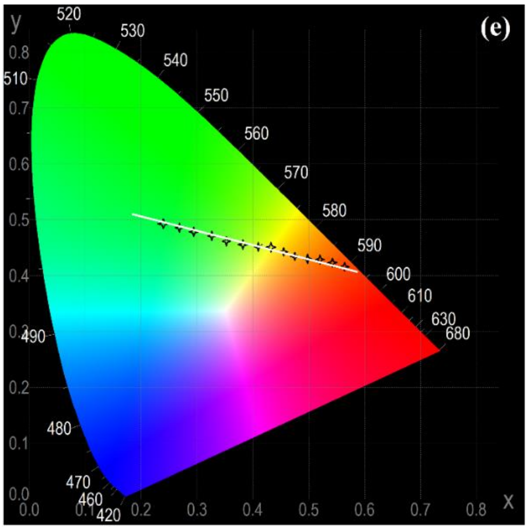

Fig. S17 The CIE chromaticity diagram, which shows the fluoresence changes of sPPDN with different concentrations of SCO-Rb aqueous solution

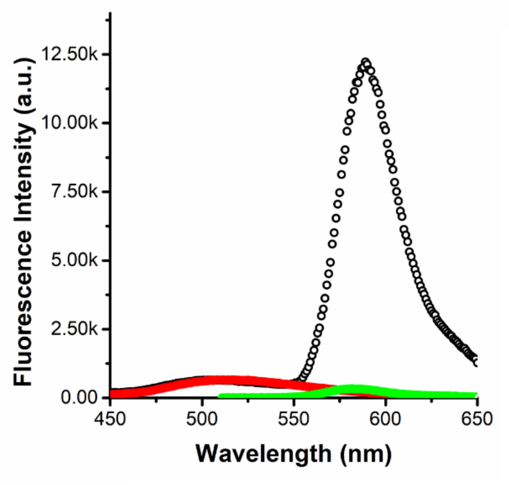

Fig. S18 Fluorescence spectra of sPPDN (D/A ratio is 2000/1) in aqueous solution, black trace (donor emission, λ_{ex} = 365 nm), green trace (acceptor emission, λ_{ex} = 580 nm). The red trace represents the fluorescence spectrum (λ_{ex} = 365 nm) of DNS, which was normalized according to the fluorescence intensity at 510 nm of the green trace

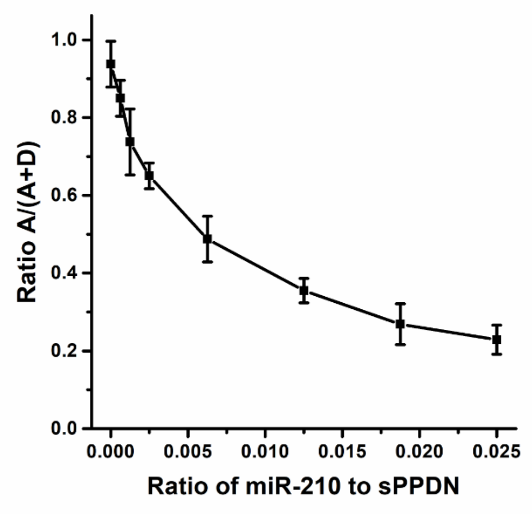

Fig. S19 Fluorescence values of FRET response A/(A + D) of sPPDN after incubation with target at different concentrations. A and D are the pick intensities of the acceptor (at 580 nm) and the donor (at 510 nm)

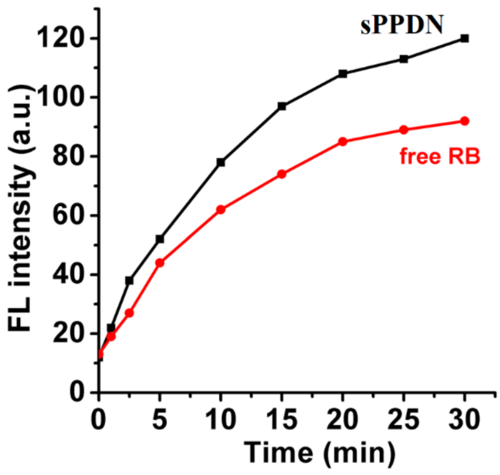

Fig. S20 ROS generation of free Rb in DMSO and sPPDN in PBS solutions laser irradiation (10 mW/cm^2) as reflected by the fluorescence intensity changes of SOSG

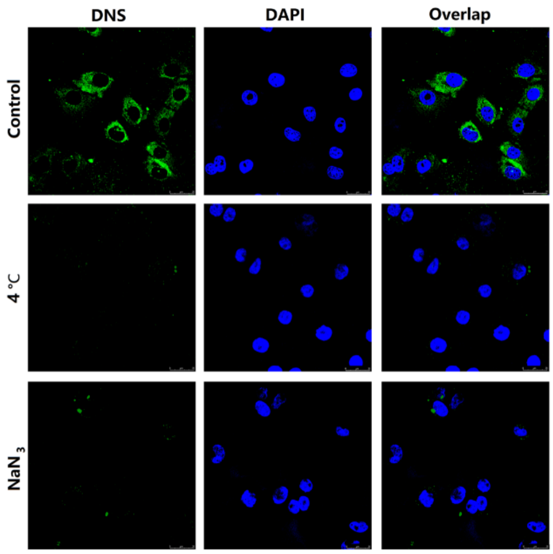

Fig. S21 Confocal fluorescence images of H1299 cells after incubation with PPDN (10 μM) under different conditions as indicated. Scale bar = 10 μm

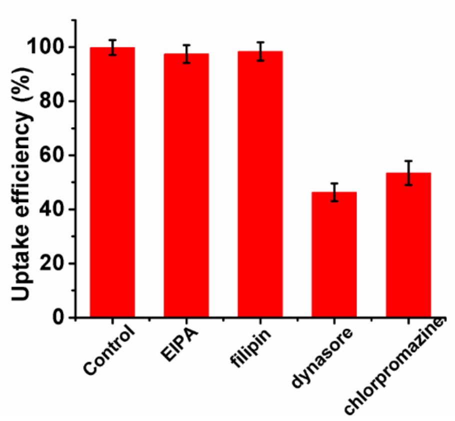

Fig. S22 Relative uptake efficiency of PPDN by H1299 cancer cells pretreated without (control) and with various endocytosis inhibitors

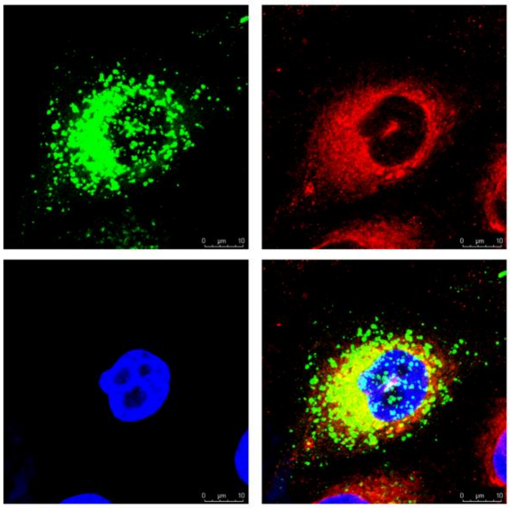

Fig. S23 Confocal fluorescence images of H1299 cancer cells stained by CellLight Early Endosomes-RFP after incubation with PPDN (10 μM) for 1 h. Scale bar = 10 μm

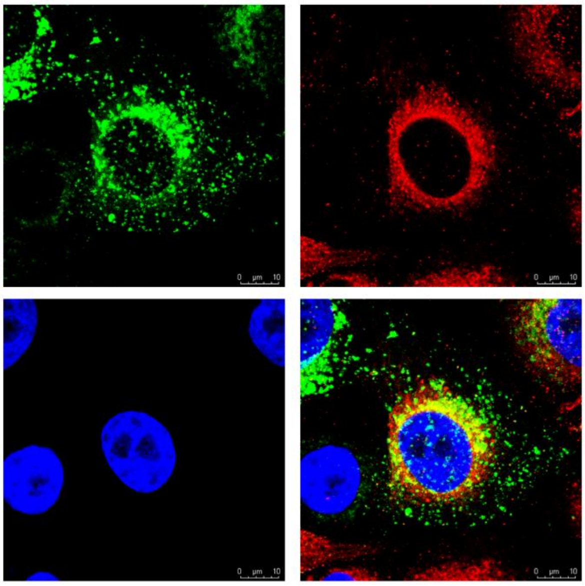

Fig. S24 Confocal fluorescence images of H1299 cancer cells stained by CellLight Late Endosomes-RFP after incubation with PPDN (10 μM) for 2 h. Scale bar = 10 μm

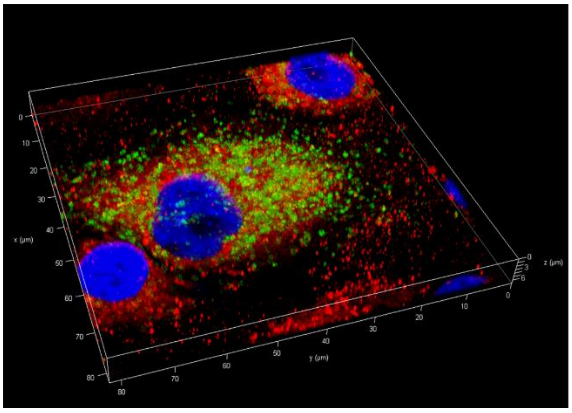

Fig. S25 Confocal fluorescence images of H1299 cancer cells stained by LysoTracker Red after incubation with PPDN (10 μM) for 4 h. Scale bar = 10 μm

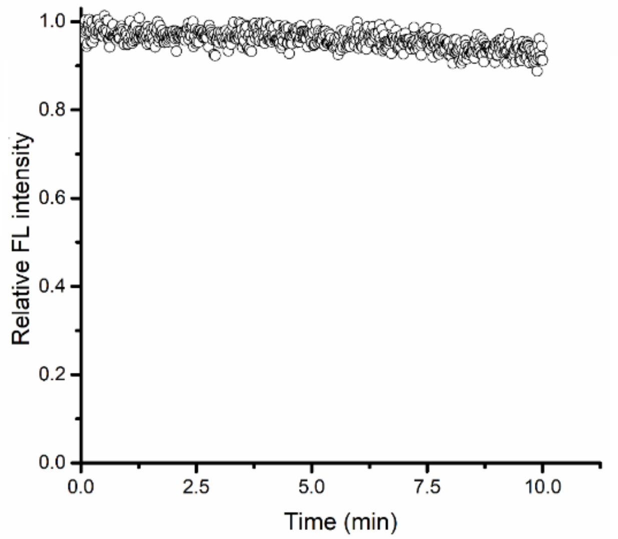

Fig. S26 The fluorescence photobleaching curves of nanoprobe irradiated for different time intervals using a 450 W xenon lamp

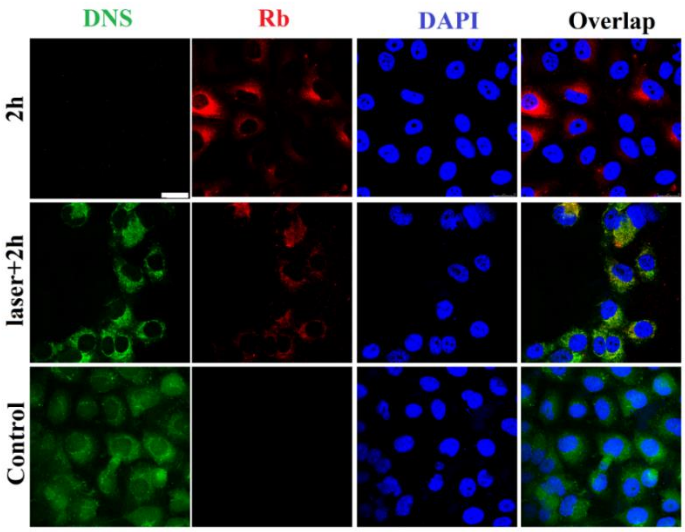

Fig. S27 Response of sPPDN to the miRNA target at the single-cell level. (**a**) Real-time fluorescent FRET images (donor channel, acceptor channels, Nucleus channel and overlay). All channels are represented at the same intensity scale. The excitation wavelength was light irradiation (10 mW/cm^2, 5 min). Signals from the donor and the acceptor were recorded at 510 and 580 nm, respectively. A and D are the integrated densities of the signal from individual particles recorded at the acceptor and the donor channels, respectively. Scale bar, 25 μm

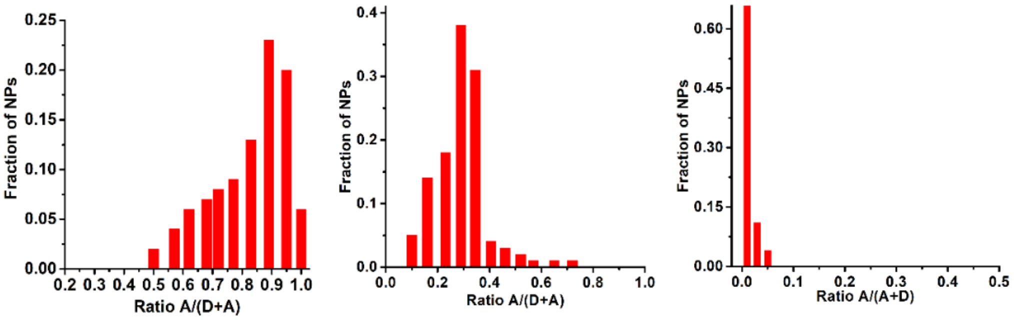

Fig. S28 The corresponding histograms of relative FRET efficiency in **Fig. S27**. A/(A + D) of the sPPDN after incubation for 2 h (first row), irradiation and incubated for another 2 h (second row) and control PPDN without DNA labeling (third row)

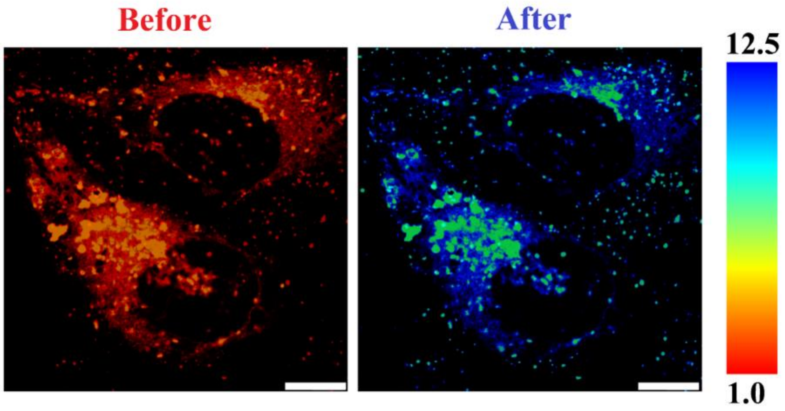

Fig. S29 FLIM-FRET measurements of living H1299 cancer cells treated with sPPDN. The FLIM images monitor the donor fluoresence lifetime before (**a**) and after photo-bleaching of the acceptor of Rb (**b**) using a 568 nm laser. Scale bar: 10 μm. The fluoresence is indicated by a false color representation. The stronger FRET process can be identified in the red color area.

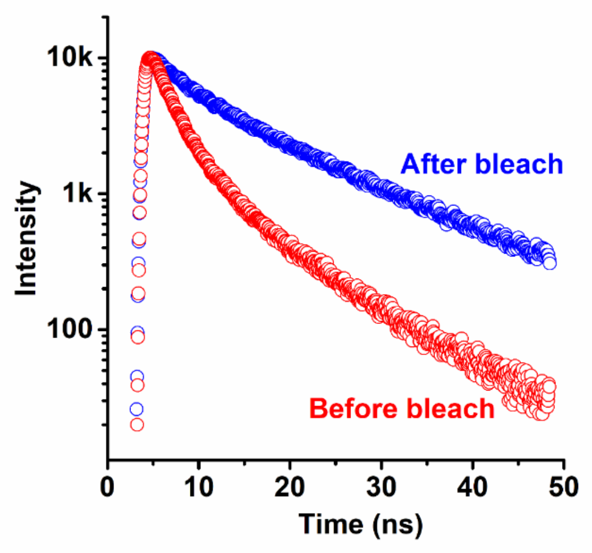

Fig. S30 The fluorescence lifetime decay curves of DNS of sPPDN before and after photo-bleaching of the acceptor of Rb. The lifetime distribution reveals the shift of the average lifetime due to FRET

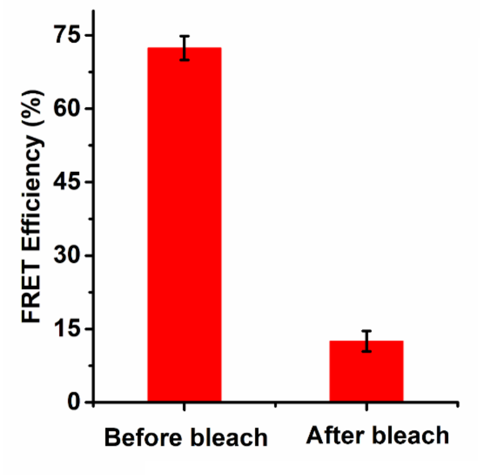

Fig. S31 FRET efficiency is shown in **Fig. S30**

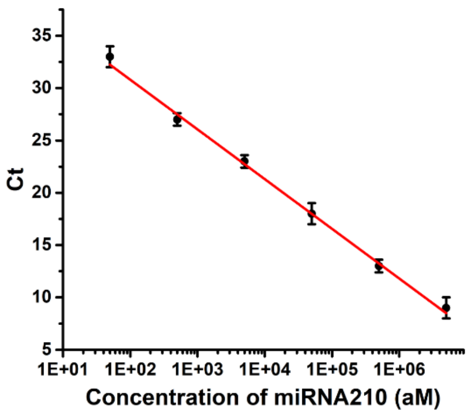

Fig. S32 Standard curve of the miR-210 in qRT-PCR. The data are presented as the mean ±3 s.d. (n = 3). Synthetic miR-210 input ranged from 50 aM to 5 × 10^6 aM in PCR

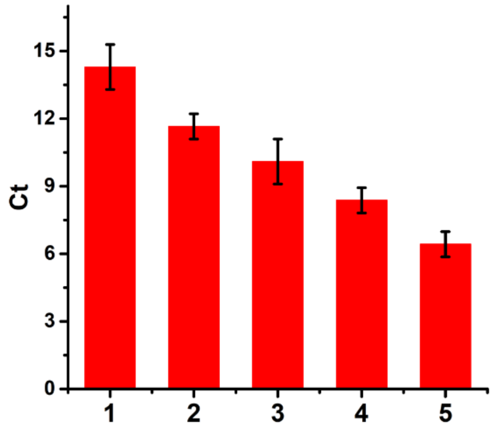

Fig. S33 qRT-PCR quantification of miR-210 in transfected H1299 cancer cells with different amounts of miR-210: Columns 1 and 2 were transfected with antisense sequences of miR-210, column 3 was not transfected, and columns 4 and 5 were transfected with both miR-210

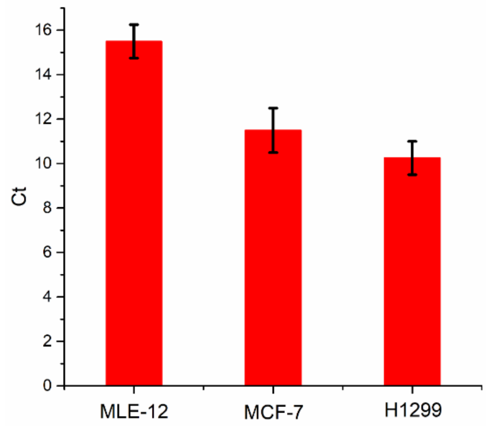

Fig. S34 qRT-PCR quantification of miR-210 in different cell lines

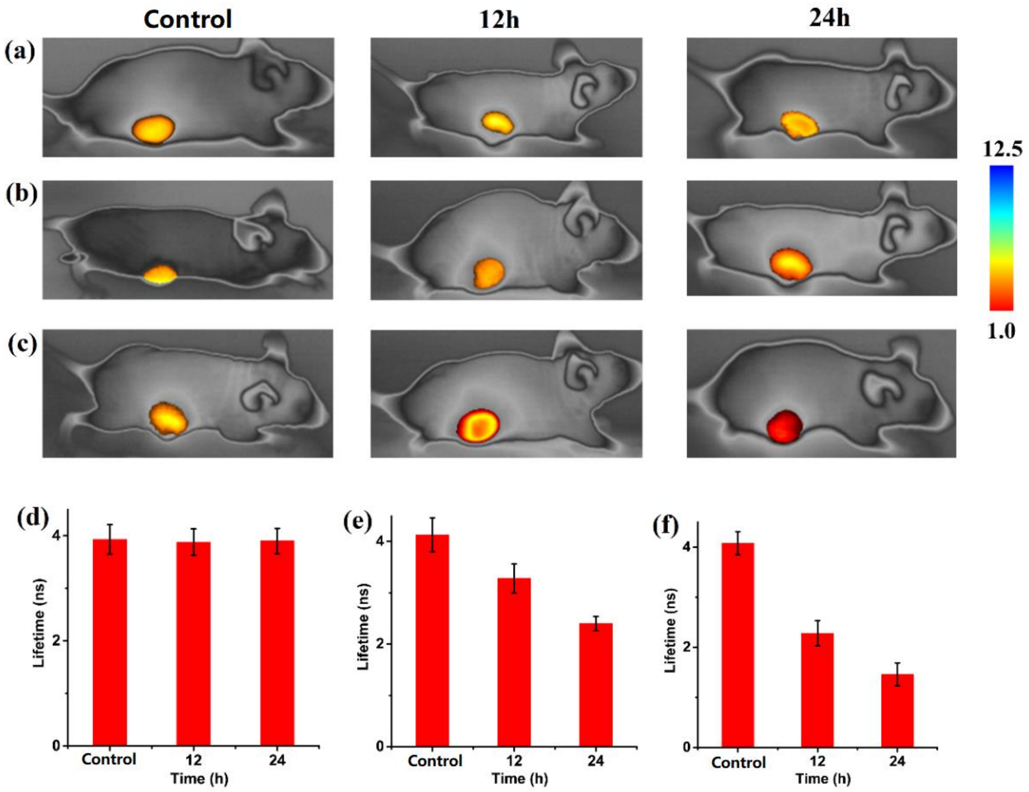

Fig. S35 (**a**) *In vivo* imaging of sPPDN for miR-210 detection. *In vivo* imaging of cancer with miR-210 markers. Ratiometric pseudocolor FLIM images of mice with different concentrations of miR-210: (**a**) 0, (**b**) 5, and (**c**) 10 mg/kg miR-210 inhibitors were injected through the tail vein, following different pre-incubation time. FLIM images were acquired under light excitation (500 mW); 588 ± 50 nm emission; exposure time 250 ms. (**d**) The relative lifetime values of mice in tumor sites with 0 mg/kg of miR-210 inhibitors treatment. (**e**) The relative lifetime values of mice in tumor sites with 5 mg/kg of miR-210 inhibitors treatment. (**f**) The relative lifetime values of mice in tumor sites with 10 mg/kg of miR-210 inhibitors treatment. Error bars are mean ± SD (n = 3 independent samples)

Table S1 Sequence of DNA and miRNA

	Sequences
miRNA-210	5'-CUGUGCGUGUGACAGCGGCUGA-3'
DBCO-labeling ssDNA-210	5'-/DBCO/TCAGCCGCTGTCACACGCACAG-3'
Rb-target-competitive sequence	5'-CUGUGCGUGUG/Rb/-3'
miRNA-210 antisense	5'-AGCCGCUGUCACACGCACAGUU-3'
miRNA-155	5'-CUCCUACAUAUUAGCAUUAACA-3'
miRNA-200	5'-UAAUACUGCCUGGUAAUGAUGA-3'
Mismatched1	5'-CUAUGCGUGUGACAGCGGCUGA-3'
Mismatched2	5'-CUGUGCGUGUGACAGCAGCUGA-3'
Mismatched3	5'-CUGUGCGUAUGACAGCGGCUGA-3'

Table S2 Time-resolved fluorescence parameters of PPD

	τ_1 (ns)	α_1 (%)	τ_2 (ns)	α_2 (%)
DNS dye	12.5	59	18.7	41
5 mol%	5.57	5	14.38	95
10 mol%	5.51	5	16.61	95
20 mol%	4.45	4	14.49	96
30 mol%	4.3	6	15.64	94
50 mol%	5.17	5	12.58	95
80 mol%	5.75	4	12.89	96
100 mol%	4.45	10.05	14.49	89.95

τ_1 : short lived decay component.

τ_2: long lived decay component.

α_1: relative contribution of the lifetime component for τ_1.

α_2: relative contribution of the lifetime component for τ_2.

The fluorescence decay may be modeled as the summation of multiple exponential components, as shown:

$$F(t) = \alpha_1 \times e^{-\frac{t}{\tau_1}} + \alpha_2 \times e^{-\frac{t}{\tau_2}}$$

Here F(t) is the measured fluorescence intensity as a function of time t; τ_i is the individual exponential components, and α_i is the coefficients of each exponential term.

Table S3 Radiative decay rates (k_r) and nonradiative decay rates (k_{nr}) of PPD and DNS dye

	K_r (10^8 S^{-1})	K_{nr} (10^8 S^{-1})
DNS dye	0.75	5.8
PPD (80 mol%)	3.56	3.3

Table S4 The fluorescence lifetime of DNS in sPPDN (DNS: 10 μM) with various concentrations of SOC-Rb (nM)

Concentration of Rb (nM)	τ_1 (ns)	α_1 (%)	τ_2 (ns)	α_2 (%)
0	5.75	4	12.89	96
0.01	6.09	28.1	12.3	71.9
0.025	4.5	38.3	12.1	61.7
0.05	3.32	43.1	12.3	56.9
0.075	3.73	52.4	11.8	47.6
0.1	2.83	60	10.3	40
0.15	2.46	67.2	9.7	32.8
0.2	2.04	76.3	8.29	23.7
0.25	1.73	79.4	7.07	20.6
0.5	1.78	88	7.82	12
0.75	1.52	91	4.95	9
1	1.5	97	3.64	3
